# Application of Newton’s polynomial interpolation scheme for variable order fractional derivative with power-law kernel

**DOI:** 10.1038/s41598-024-66494-z

**Published:** 2024-07-12

**Authors:** S Naveen, V Parthiban

**Affiliations:** Department of Mathematics, School of Advanced Sciences, Vellore Institute of Technology, Chennai Campus, Chennai, 600127 India

**Keywords:** Atangana–Toufik scheme, Chaotic system, Variable order fractional derivative, Rikitake system, Rucklidge system, Wang–Sun system, Engineering, Mathematics and computing, Physics

## Abstract

This paper, offers a new method for simulating variable-order fractional differential operators with numerous types of fractional derivatives, such as the Caputo derivative, the Caputo–Fabrizio derivative, the Atangana–Baleanu fractal and fractional derivative, and the Atangana–Baleanu Caputo derivative via power-law kernels. Modeling chaotical systems and nonlinear fractional differential equations can be accomplished with the utilization of variable-order differential operators. The computational structures are based on the fractional calculus and Newton’s polynomial interpolation. These methods are applied to different variable-order fractional derivatives for Wang–Sun, Rucklidge, and Rikitake systems. We illustrate this novel approach’s significance and effectiveness through numerical examples.

## Introduction

Since traditional or classical differentiation has developed into the domain of local operators, advances in differentiation and integration have occurred. After the classical derivative was convolutioned with a kernel of the power-law kind, fractional calculus emerged as a new calculus. This novel calculus has been applied in several scientific fields during the last few decades, with significant results demonstrated by innovative results and the publication of academic studies. Studying fractional modeling of chaotic systems holds significant importance because traditional integer-order differential equations often struggle to capture the memory effects present in real-world chaotic phenomena. Fractional derivatives, with their ability to represent non-local memory, offer a more nuanced approach. This allows for more accurate modeling of complex dynamics in various fields, from electrical circuits and biological systems to financial markets, leading to better predictions and control strategies for these chaotic systems.

Riemann-Liouville and Caputo derivatives are two different kinds of fractional differential and integral operators that are known in fractional calculus (see in^1–4^). The initial one is a power law convolution with the first derivative, while the later includes the Caputo–Fabrizio fractional derivative, which is an exponential decay law convolution with the first derivative that has a Delta-Dirac feature. The convolution of the generalized Mittag-Leffler function with the first derivative is the third kind, also referred to as the Atangana–Baleanu fractional derivative. These fractional-order derivative operators have been successfully investigated in a wide range of scientific applications. These applications include a broad spectrum of fractional calculus models with operators based on these three kinds of kernels in^5^. A novel concept of fractional derivative without singular kernel was studied by Caputo et al. ^6^.

However it has been used in a number of situations, this specific differential operator has not received much recognition in the fields of science, technology, and engineering. Nevertheless, the appearance of this operator with a fractal derivative occurs in many natural processes. Its usefulness in defining preferred routes for groundwater conveyance serves as an example. While some adhere to the notion that there is nothing entirely new under the sun, it becomes evident, or at least debatable, that combining two preexisting elements can yield a novel and inventive concept or, at the very least, a modified iteration of earlier ideas. When compared to known principles, these alterations could potentially improve efficiency and application. Analytical techniques might not be successful in providing suggestions since models are non-linear. Numerical solutions are required in situations where conventional analytical methods are inadequate. Thus, our goal is to create a novel numerical system that can solve integral and differential equations, both linear and non-linear, efficiently. A number of investigations demonstrate that a Newton’s polynomial is more accurate than a Lagrange polynomial. As such, the numerical technique we suggest is based on the use of a Newton’s polynomial. The authors discussed Newton’s polynomial interpolations in various scenarios in the distinct systems in^7–10^.

Variable-order fractional derivatives (VOFDEs) offer a significant advantage over traditional fractional differential equations (FDEs) by allowing the order of differentiation to change within the equation itself. This added flexibility makes VOFDEs better suited for modeling real-world phenomena where the memory or hereditary effects influencing the system dynamics aren’t constant, leading to more accurate and nuanced descriptions of complex systems in physics, engineering, and biology. Recently, VOFDEs have played a major role in real-life scenarios. Ramirez et al. ^11^ investigated the variable-order constitutive relation for viscoelasticity. Sun et al. ^12^ discussed the variable-order fractional differential operators in anomalous diffusion modeling. Chauhan et al. studied a validation of the concept of a formula for variable-order integrals and derivatives in^13^. Sun et al. ^14^ study a review of variable-order fractional differential equations: mathematical foundations, physical models, numerical methods, and applications. Shichang et al. ^15^ investigate the numerical solutions of a variable-order fractional financial system. Naveen et al. ^16^ study the existence and uniqueness of variable-order fractional derivatives with time-independent delays. The sliding mode control plays a major role in chaos theory for examples^17–19^. Moreover, the authors discussed the three dimensional Lotka-Volterra attractor for the fractional order control strategy in^20^. In^21^, the authors investigated the generalized invexity and duality in multi-objective various problems involving non-singular fractional derivative.

The authors examined Newton’s polynomial, a novel numerical technique for solving ordinary differential equations, in^22^. The authors of^23^ addressed secret sharing for multi-factor authentication using Newton’s polynomial. Gutama et al. ^24^ studied Newton’s polynomial-based automatic model generation for sensor calibration to enhance the functionality of an inexpensive ultrasonic range finder. Furthermore, as can be discovered in^25–28^, Newton’s polynomial interpolations are employed in several real-world situations, such as the Ginzburg-Landau equation, Burke-Shaw systems, as well as non-local and non-singular kernels, etc. The authors of^29^ examined systems of nonlinear differential equations with variable coefficients and their Newton-Taylor polynomial solutions. Sara Dadras et al. analyzed a unique three-dimensional autonomous chaotic system that produced two, three, and four-scroll attractors in^30^. The authors examined a three-dimensional four-wing attractor and its analysis in^31^. In^32^, the authors discussed a new numerical scheme based on Newton polynomial with application to fractional nonlinear differential equations. In^33^, the authors investigated the various chaotic dynamical system of fractal and fractional derivative with using the new numerical schemes. In^34,35^ the authors investigated the Hilfer-Prabhakar fractional derivative for some integral transform results and diffusion equations. In^36^ the authors analyzed the food chain model with disease in intermediate predator. Fractal dynamics and computational analysis of local fractional Poisson equations in electrostatics, the Fokker-Planck equations in Brownian motions, and partial differential equations are studied in^37–39^. In^40^ the author investigated the chaos in the Rikitake two-disc dynamo system and Rikitake systems plays a major role in various scenarios such as^41,42^. In^43^ the authors studied a application in secured communication using OFDM and its controlling with a single controller using linear feedback and passive control methods in^44^.

The primary contribution of this endeavor can be outlined as follows:

1. The solution to the initial value problem for variable-order fractional derivatives has been achieved through the Newton’s polynomial interpolation schemes with application of the power law kernel.
2. Recently, in^45^, the authors discussed the variable-order fractional financial system and memcapacitor-based circuit system of various derivatives with two-step Lagrange polynomial approximation.
3. We apply the new numerical approximation for the various aperiodic systems of various variable-order fractional derivatives, such as the Caputo derivative, the Caputo–Fabrizio derivative, the Atangana–Baleanu fractal and fractional derivative, and the Atangana–Baleanu Caputo derivative.
4. The distinct variable-order derivatives are applied to the various aperiodic systems, such as the Wang–Sun, Rucklidge, and Rikitake systems applied for Newton’s polynomial interpolation scheme.

The paper is organized as follows: the basic definitions of variable-order fractional derivatives are studied in Section “Preliminaries”. In Section “New numerical approach”, see about the new numerical scheme of variable order derivative. Various fractional derivatives of variable order are investigated in Section “Variable order fractional derivative”, and some validation of the numerical schemes is presented. In Section “Stability analysis”, study about the stability analysis of Rikitake dynamical system. In Section “Comparative study”, there is a there is a comparison study about the four distinct variable-order fractional derivatives. Finally, the conclusion is described in the last section.

## Preliminaries

In this section the basic definition of variable order distinct fractional derivatives are discussed.

### Definition 1.1

^5^ The Caputo fractional derivative with variable order $\alpha (\tau )$ is defined as

$$\begin{aligned} {}^C{\mathbb {D}}^{\alpha (\tau )}g(\tau )=\frac{1}{\Gamma (m-\alpha (\tau ))}\int \limits _0^\tau (\tau -t)^{m-\alpha (\tau )-1}g^{(m)}(t)dt,\quad m-1<\alpha (\tau )\le m, \end{aligned}$$

where $\Gamma (\alpha (\tau ))$ is Gamma function.

### Definition 1.2

^5^ The Caputo–Fabrizio derivative with variable order $\alpha (\tau )$ is defined by

$$\begin{aligned} {}^{CF}{\mathbb {D}}^{\alpha (\tau )}g(\tau )=\frac{(2-\alpha (\tau ))M(\alpha (\tau ))}{2(1-\alpha (\tau ))}\int \limits _0^\tau g(t)\exp \left[ -\frac{\alpha (\tau )}{1-\alpha (\tau )}(\tau -t)\right] dt, \end{aligned}$$

where $M(\alpha (\tau ))$ is normalization function such that $M(0)=M(1)=1$.

### Definition 1.3

^5^ The Atangana–Baleanu–Caputo fractional variable order derivative with order $\alpha (\tau )$ is defined by

$$\begin{aligned} {}^{ABC}{\mathbb {D}}^{\alpha (\tau )}g(\tau )=\frac{AB(\alpha (\tau ))}{1-\alpha (\tau )}\int \limits _0^\tau g'(t){\mathbb {E}}_{\alpha (\tau )}\left[ -\frac{\alpha (\tau )}{1-\alpha (\tau )}(\tau -t)^{\alpha (\tau )}\right] dt. \end{aligned}$$

### Definition 1.4

^5^ The Atangana–Baleanu fractal-fractional derivative with Caputo sense variable order fractional derivative with order $\alpha (\tau )$ is defined by

$$\begin{aligned} {}^{ABFF}{\mathbb {D}}^{\alpha ,\beta (\tau )}g(\tau )=\frac{AB(\alpha )}{1-\alpha }\frac{d}{d\tau ^{\beta (\tau )}}\int \limits _0^\tau g(t){\mathbb {E}}_\alpha \left[ -\frac{\alpha }{1-\alpha }(\tau -t)^\alpha \right] g(t)\ dt, \end{aligned}$$

where $$AB(\alpha )=1-\alpha +\frac{\alpha }{\Gamma (\alpha )}$$.

## New numerical approach

In this section, let us discuss the new numerical scheme for variable-order fractional derivatives.

Recently, Atangana and Seda proposed an alternative numerical approach grounded in the Newton’s polynomial. The adoption of this method was warmly embraced, proving to be a proficient and precise numerical scheme capable of addressing fractional ordinary differential equations and fractional systems of ordinary differential equations featuring various types of fractional integral operators.

### Caputo fractional derivative

This subsection talks about the generalized Newton’s two-step polynomial approximation of the variable-order Caputo fractional derivative.

Consider the general Cauchy problem of Caputo derivative,

$$\begin{aligned} {}^C{\mathbb {D}}^{\alpha (\tau )}x(\tau )=g(\tau ,x(\tau ))\ \text {with}\ x(0)=x_0. \end{aligned}$$

By applying the definition of Caputo derivative

$$\begin{aligned} x(\tau )=x(0)+\frac{1}{\Gamma (\alpha (\tau ))}\int \limits _0^\tau (\tau -t)^{\alpha (\tau )-1}g(t,x(t))dt. \end{aligned}$$

Which implies that $\tau _{p+1}=(p+1)h$, where $p=0,1,2,\cdots .$

$$\begin{aligned} x(\tau _{p+1})=x(0)+\frac{1}{\Gamma (\alpha (\tau ))}\int \limits _0^{\tau _{p+1}}(\tau -t)^{\alpha (\tau )-1}g(t,x(t))dt, \end{aligned}$$

by applying the Newton’s polynomial,

1 $$\begin{aligned} x_{p+1}=&x_0+\frac{1}{\Gamma (\alpha (\tau ))}\sum \limits _{n=2}^p\int \limits _{\tau _n}^{\tau _{n+1}} \begin{Bmatrix} g\left( \tau _{n-2}, x_{n-2}\right) \\ +\frac{1}{h}\left( g\left( \tau _{n-1}, x_{n-1}\right) -g\left( \tau _{n-2}, x_{n-2}\right) \right) \left( t-\tau _{n-2}\right) \\ +\frac{1}{2h^2}g\left( \tau _n, x_n\right) -2 g\left( \tau _{n-1}, x_{n-1}\right) +g\left( \tau _{n-2}, x_{n-2}\right) \\ \times \left( t-\tau _{n-2}\right) \left( t-\tau _{n-1}\right) \end{Bmatrix} (\tau _{p+1}-t)^{\alpha (\tau )-1}dt.\nonumber \\ x_{p+1}=&x_0+\frac{h^{\alpha (\tau )}}{\Gamma (\alpha (\tau )+1)} \sum \limits _{n=2}^p g\left( \tau _{n-2}, x_{n-2}\right) \left[ (p-n+1)^{\alpha (\tau )}-(p-n)^{\alpha (\tau )}\right] \nonumber \\&+\frac{h^{\alpha (\tau )}}{\Gamma (\alpha (\tau )+2)} \sum \limits _{n=2}^p\left[ g\left( \tau _{n-1}, x_{n-1}\right) -g\left( \tau _{n-2}, x_{n-2}\right) \right] \times A_1\nonumber \\&+\frac{h^{\alpha (\tau )}}{2 \Gamma (\alpha (\tau )+3)} \sum \limits _{n=2}^p\left[ g\left( \tau _n, x_n\right) -2g\left( \tau _{n-1}, x_{n-1}\right) +g\left( \tau _{n-2}, x_{n-2}\right) \right] \times A_2. \end{aligned}$$

where $$A_1=\begin{array}{c} (p-n+1)^{\alpha (\tau )}(p-n+3+2 \alpha (\tau ))-(p-n)^{\alpha (\tau )}(p-n+3+3 \alpha (\tau )) \end{array}$$ and $$A_2=\left[ \begin{array}{c} (p-n+1)^{\alpha (\tau )}\left( \begin{array}{c} 2(p-n)^2+(3 \alpha (\tau )+10)(p-n)+2 \alpha (\tau )^2+9 \alpha (\tau )+12 \end{array}\right) \\ -(p-n)^{\alpha (\tau )}\left( \begin{array}{c} 2(p-n)^2+(5 \alpha (\tau )+10)(p-n) +6 \alpha (\tau )^2+18 \alpha (\tau )+12 \end{array}\right) \end{array}\right]$$.

### Caputo–Fabrizio fractional derivative

Consider the general Cauchy problem of Caputo–Fabrizio derivative,

$$\begin{aligned} {}^{CF}{\mathbb {D}}^{\alpha (\tau )}x(\tau )=g(\tau ,x(\tau ))\ \text {with}\ x(0)=x_0. \end{aligned}$$

By applying the definition of Caputo–Fabrizio integral,

$$\begin{aligned} x(\tau )=x(0)+\frac{1-\alpha (\tau )}{M(\alpha (\tau ))}g(\tau ,x(\tau ))+\frac{\alpha (\tau )}{M(\alpha (\tau ))}\int \limits _0^\tau g(t,x(t))dt. \end{aligned}$$

Which implies that $\tau _{p+1}=(p+1)h$,

$$\begin{aligned} x\left( \tau _{p+1}\right) -x(0)=\frac{1-\alpha (\tau )}{M(\alpha (\tau ))} g\left( \tau _p, x\left( \tau _p\right) \right) +\frac{\alpha (\tau )}{M(\alpha (\tau ))} \int \limits _0^{\tau _{p+1}} g(t, x(t)) dt, \end{aligned}$$

at the point $\tau _p=ph$, we have

$$\begin{aligned} x\left( \tau _p\right) -x(0)=\frac{1-\alpha (\tau )}{M(\alpha (\tau ))} g\left( \tau _{p-1}, x\left( \tau _{p-1}\right) \right) +\frac{\alpha (\tau )}{M(\alpha (\tau ))} \int \limits _0^{\tau _p} g(t, x(t)) dt, \end{aligned}$$

which implies that,

$$\begin{aligned} x\left( \tau _{p+1}\right) -x\left( \tau _p\right)&=\frac{1-\alpha (t)}{M(\alpha (\tau ))}\left[ g\left( \tau _p, x\left( \tau _p\right) \right) -g\left( \tau _{p-1}, x\left( _{p-1}\right) \right) \right] +\frac{\alpha (\tau )}{M(\alpha (\tau ))} \int \limits _{\tau _p}^{\tau _{p+1}} g(t, x(t)) dt. \end{aligned}$$

By applying the Newton’s polynomial approximation, we get,

$$\begin{aligned} x^{p+1}-x^p=&\frac{1-\alpha (\tau )}{M(\alpha (\tau ))}\left[ g\left( \tau _p, x\left( \tau _p\right) \right) -g\left( \tau _{p-1}, x\left( \tau _{p-1}\right) \right) \right] \\&+\frac{\alpha (\tau )}{M(\alpha (\tau ))} \int \limits _{\tau _p}^{\tau _{p+1}}\left\{ \begin{array}{c} g\left( \tau _{p-2}, x^{p-2}\right) \\ +\frac{g\left( \tau _{p-1},x^{p-1}\right) -g\left( \tau _{p-2}, x^{p-2}\right) }{h}\left( t-\tau _{p-2}\right) \\ +\frac{g\left( \tau _p, x^p\right) -2 g\left( \tau _{p-1}, x^{p-1}\right) +g\left( \tau _{p-2}, x^{p-2}\right) }{2h^2} \\ \times \left( t-\tau _{p-2}\right) \left( t-\tau _{p-1}\right) \end{array}\right\} dt, \end{aligned}$$

and write it as follows:

$$\begin{aligned} x^{p+1}-x^p=&\frac{1-\alpha (\tau )}{M(\alpha (\tau ))}\left[ g\left( \tau _p, u\left( \tau _p\right) \right) -g\left( \tau _{p-1}, x\left( \tau _{p-1}\right) \right) \right] \\&+\frac{\alpha (\tau )}{M(\alpha (\tau ))}\left\{ \begin{array}{c} g\left( \tau _{p-2}, x^{p-2}\right) h \\ +\frac{g\left( \tau _{p-1}, x^{p-1}\right) -g\left( \tau _{p-2}, x^{p-2}\right) }{h} \int \limits _{\tau _p}^{\tau _{p+1}}\left( t-\tau _{p-2}\right) dt \\ +\frac{g\left( \tau _p, x^p\right) -2 g\left( \tau _{p-1}, x^{p-1}\right) +g\left( \tau _{p-2}, x^{p-2}\right) }{2h^2} \\ \times \int \limits _{\tau _p}^{\tau _{p+1}}\left( t-\tau _{p-2}\right) \left( t-\tau _{p-1}\right) dt \end{array}\right\} . \\&\end{aligned}$$

Then we can rearrange the above equation and we get the two step Newton’s approximation approach,

2 $$\begin{aligned} x^{p+1}=&x^p+\frac{1-\alpha (\tau )}{M(\alpha (\tau ))}\left[ g\left( \tau _p, x\left( \tau _p\right) \right) -g\left( \tau _{p-1}, x\left( \tau _{p-1}\right) \right) \right] +\frac{\alpha (\tau )}{M(\alpha (\tau ))}\left\{ \begin{array}{c} \frac{23}{12} g\left( \tau _p, x^p\right) h-\frac{4}{3} g\left( \tau _{p-1}, x^{p-1}\right) h\\ +\frac{5}{12} g\left( \tau _{p-2}, x^{p-2}\right) . \end{array}\right\} . \end{aligned}$$

### Atangana–Baleanu–Caputo fractional derivative

Consider the general Cauchy problem of Atangana–Baleanu–Caputo derivative,

$$\begin{aligned} {}^{ABC}{\mathbb {D}}^{\alpha (\tau )}x(\tau )=g(\tau ,x(\tau ))\ \text {with}\ x(0)=x_0. \end{aligned}$$

By applying the definition of Atangana–Baleanu integral,

$$\begin{aligned} x(\tau )=x(0)+\frac{1-\alpha (\tau )}{AB(\alpha (\tau ))}g(\tau ,x(\tau ))+\frac{\alpha (\tau )}{AB(\alpha (\tau ))\Gamma (\alpha (\tau ))}\int \limits _0^\tau g(\tau ,x(\tau ))(\tau -t)^{\alpha (\tau )-1}dt. \end{aligned}$$

Then,

$$\begin{aligned} x(\tau _{p+1})=x(0)+\frac{1-\alpha (\tau )}{AB(\alpha (\tau ))}g(\tau ,x(\tau ))+\frac{\alpha (\tau )}{AB(\alpha (\tau ))\Gamma (\alpha (\tau ))}\int \limits _0^{\tau _{p+1}}g(\tau ,x(\tau ))(\tau _{p+1}-t)^{\alpha (\tau )-1}dt, \end{aligned}$$

we can reform,

$$\begin{aligned} x(\tau _{p+1})=x(0)+\frac{1-\alpha (\tau )}{AB(\alpha (\tau ))}g(\tau _p,x^p)+\frac{\alpha (\tau )}{AB(\alpha (\tau ))\Gamma (\alpha (\tau ))}\sum \limits _{n=2}^p\int \limits _{t_n}^{\tau _{n+1}}g(\tau ,x(\tau ))(\tau _{p+1}-t)^{\alpha (\tau )-1}dt, \end{aligned}$$

by applying the Newton’s polynomial of the above equation, we get

$$\begin{aligned} x^{p+1}=&x(0)+\frac{1-\alpha (\tau )}{A B(\alpha (\tau ))} g\left( \tau _p, x^p\right) +\frac{\alpha (\tau )}{A B(\alpha (\tau )) \Gamma (\alpha (\tau ))} \\&\times \sum \limits _{n=2}^p \int \limits _{\tau _n}^{\tau _{n+1}}\left\{ \begin{array}{c} g\left( \tau _{n-2}, x^{n-2}\right) \\ +\frac{g\left( \tau _{n-1}, x^{n-1}\right) -g\left( \tau _{n-2}, x^{n-2}\right) }{h} \\ \times \left( t-\tau _{n-2}\right) \\ +\frac{g\left( \tau _n, x^n\right) -2 g\left( \tau _{n-1}, x^{n-1}\right) +g\left( \tau _{n-2}, x^{n-2}\right) }{2h^2} \\ \times \left( t-\tau _{n-2}\right) \left( t-\tau _{n-1}\right) \end{array}\right\} \times \left( \tau _{p+1}-t\right) ^{\alpha (\tau )-1} dt .\\ x^{p+1} =&x(0)+\frac{1-\alpha (\tau )}{A B(\alpha (\tau ))} g\left( \tau _p, x^p\right) +\frac{\alpha (\tau )}{A B(\alpha (\tau )) \Gamma (\alpha (\tau ))} \sum \limits _{n=2}^p g\left( \tau _{n-2}, x^{n-2}\right) \int \limits _{\tau _n}^{\tau _{n+1}}\left( \tau _{p+1}-t\right) ^{\alpha (\tau )-1} dt\\&+\frac{\alpha (\tau )}{A B(\alpha (\tau )) \Gamma (\alpha (\tau ))} \sum \limits _{n=2}^p \frac{g\left( \tau _{n-1}, x^{n-1}\right) -g\left( \tau _{n-2}, x^{n-2}\right) }{h}\int \limits _{\tau _n}^{\tau _{n+1}}\left( t-\tau _{n-2}\right) \left( \tau _{p+1}-t\right) ^{\alpha (\tau )-1} dt\\&+\frac{\alpha (\tau )}{A B(\alpha (\tau )) \Gamma (\alpha (\tau ))} \sum \limits _{n=2}^p \frac{g\left( \tau _n, x^n\right) -2 g\left( \tau _{n-1}, x^{n-1}\right) +g\left( \tau _{n-2}, x^{n-2}\right) }{2h^2}\\&\int \limits _{\tau _n}^{\tau _{n+1}}\left( t-\tau _{n-2}\right) \left( t-\tau _{n-1}\right) \left( \tau _{p+1}-t\right) ^{\alpha (\tau )-1} dt. \end{aligned}$$

Then we can rearrange the above equation and we get the two step Newton’s approximation approach

3 $$\begin{aligned} x^{p+1}=&x(0)+\frac{1-\alpha (\tau )}{A B(\alpha (\tau ))} g\left( \tau _p, x^p\right) \nonumber \\&+\frac{\alpha (\tau )h^{\alpha (\tau )}}{A B(\alpha (\tau )) \Gamma (\alpha (\tau )+1)} \sum \limits _{n=2}^p g\left( \tau _{n-2}, x^{n-2}\right) \left[ (p-n+1)^{\alpha (\tau )}-(p-n)^{\alpha (\tau )}\right] \nonumber \\&+\frac{\alpha (\tau )h^{\alpha (\tau )}}{A B(\alpha (\tau )) \Gamma (\alpha (\tau )+2)} \sum \limits _{n=2}^p\left[ g\left( \tau _{n-1}, x^{n-1}\right) -g\left( \tau _{n-2}, x^{n-2}\right) \right] \times A_3\\&+\frac{\alpha (\tau )h^{\alpha (\tau )}}{2 A B(\alpha (\tau )) \Gamma (\alpha (\tau )+3)} \sum \limits _{n=2}^p\left[ \begin{array}{c} g\left( \tau _n, x^n\right) -2 g\left( \tau _{n-1}, x^{n-1}\right) \\ \nonumber +g\left( \tau _{n-2}, x^{n-2}\right) \end{array}\right] \times A_4, \end{aligned}$$

where $$A_3=\left[ \begin{array}{c} (p-n+1)^{\alpha (\tau )}(p-n+3+2 \alpha (\tau ))-(p-n)^{\alpha (\tau )}(p-n+3+3 \alpha (\tau )) \end{array}\right]$$ and $$A_4=\left[ \begin{array}{c} (p-n+1)^{\alpha (\tau )}\left( \begin{array}{c} 2(p-n)^2+(3 \alpha (\tau )+10)(p-n) +2 \alpha (\tau )^2+9 \alpha (\tau )+12 \end{array}\right) \\ -(p-n)^{\alpha (\tau )}\left( \begin{array}{c} 2(p-n)^2+(5 \alpha (\tau )+10)(p-n)+6 \alpha (\tau )^2+18 \alpha (\tau )+12 \end{array}\right) \end{array}\right]$$.

### Atangana–Baleanu–Fractal fractional derivative

Consider the general Cauchy problem of Atangana–Baleanu fractal fractional derivative

$$\begin{aligned} {}^{ABFF}{\mathbb {D}}^{\alpha ,\beta (\tau )}x(\tau )=g(\tau ,x(\tau ))\ \text {with}\ x(0)=x_0. \end{aligned}$$

By applying the definition of fractal fractional integral,

$$\begin{aligned} x(\tau )=&x(0)+\frac{1-\alpha }{AB(\alpha )}\tau ^{\beta (\tau )}\left[ \beta '(\tau )\ln (\tau )+\frac{\beta (\tau )}{\tau }\right] g(\tau ,x(\tau ))\\&+\frac{\alpha }{AB(\alpha )\Gamma (\alpha )}\int \limits _0^\tau g(t,x(t))(\tau -t)^{\alpha -1}\left[ \beta '(t)\ln (t)+\frac{\beta (t)}{t}\right] t^{\beta (t)}dt. \end{aligned}$$

Then,

$$\begin{aligned} x\left( \tau _{p+1}\right) =x(0)&+\frac{1-\alpha }{A B(\alpha )} \tau _p^{\beta \left( \tau _p\right) }\left[ \frac{\beta \left( \tau _{p+1}\right) -\beta \left( \tau _p\right) }{h} \ln \tau _p+\frac{\beta \left( \tau _p\right) }{\tau _p}\right] g\left( \tau _p, x\left( \tau _p\right) \right) \\&+\frac{\alpha }{A B(\alpha ) \Gamma (\alpha )} \int \limits _0^{\tau _{p+1}} g(t, x(t))\left( \tau _{p+1}-t\right) ^{\alpha -1}\left[ \beta '(t)\ln (t)+\frac{\beta (t)}{t}\right] t^{\beta (t)}dt, \end{aligned}$$

let us consider for our convenience,

$$\begin{aligned} G(t,x(t))=&g(t,x(t))\left[ \beta '(t)\ln (t)+\frac{\beta (t)}{t}\right] t^{\beta (t)}\\ x\left( \tau _{p+1}\right) =&x(0)+\frac{1-\alpha }{A B(\alpha )} \tau _p^{\beta \left( \tau _p\right) }\left[ \frac{\beta \left( \tau _{p+1}\right) -\beta \left( \tau _p\right) }{h} \ln \tau _p+\frac{\beta \left( \tau _p\right) }{\tau _p}\right] g\left( \tau _p, x\left( \tau _p\right) \right) \\&+\frac{\alpha }{A B(\alpha ) \Gamma (\alpha )} \sum \limits _{n=2}^p \int \limits _{\tau _n}^{\tau _{n+1}} G(t, x(t))\left( \tau _{p+1}-t\right) ^{\alpha -1} d t. \end{aligned}$$

Applying the Newton’s polynomial approximation, we get

4 $$\begin{aligned} x^{p+1} =&x(0)+\frac{1-\alpha }{A B(\alpha )} \tau _p^{\beta \left( \tau _p\right) }\left[ \frac{\beta \left( \tau _{p+1}\right) -\beta \left( \tau _p\right) }{h} \ln \tau _p+\frac{\beta \left( \tau _p\right) }{\tau _p}\right] g\left( \tau _p, x\left( \tau _p\right) \right) \nonumber \\&+\frac{\alpha }{A B(\alpha ) \Gamma (\alpha )}\times \sum \limits _{n=2}^p \int \limits _{\tau _n}^{\tau _{n+1}}\left\{ \begin{array}{c} G\left( \tau _{n-2}, x\left( \tau _{n-2}\right) \right) \\ +\frac{G\left( \tau _{n-1}, x\left( \tau _{n-1}\right) \right) -G\left( \tau _{n-2}, x\left( \tau _{n-2}\right) \right) }{h} \\ \times \left( t-\tau _{n-2}\right) \\ +\frac{G\left( \tau _n, x\left( \tau _n\right) \right) -2 G\left( \tau _{n-1}, x\left( \tau _{n-1}\right) \right) +G\left( \tau _{n-2}, x\left( \tau _{n-2}\right) \right) }{2h^2} \\ \times \left( t-\tau _{n-2}\right) \left( t-\tau _{\tau -1}\right) \end{array}\right\} (\tau _{p+1}-t)^{\alpha -1}dt,\nonumber \\ x^{p+1} =&x(0)+\frac{1-\alpha }{A B(\alpha )} \tau _p^{\beta \left( \tau _p\right) }\left[ \frac{\beta \left( \tau _{p+1}\right) -\beta \left( \tau _p\right) }{h} \ln \tau _p+\frac{\beta \left( \tau _p\right) }{\tau _p}\right] g\left( \tau _p, x\left( \tau _p\right) \right) \nonumber \\&+\frac{\alpha }{A B(\alpha )} \frac{h^\alpha }{\Gamma (\alpha +1)} \sum \limits _{n=2}^p \tau _{p-2}^{\beta \left( \tau _{n-2}\right) }\left[ \frac{\beta \left( \tau _{n-1}\right) -\beta \left( \tau _{n-2}\right) }{h} \ln \tau _{n-2}+\frac{\beta \left( \tau _{n-2}\right) }{\tau _{n-2}}\right] \nonumber \\&\times g\left( \tau _{n-2}, x\left( \tau _{n-2}\right) \right) \left[ (p-n+1)^\alpha -(p-n)^\alpha \right] \nonumber \\&+\frac{\alpha }{A B(\alpha )} \frac{h^\alpha }{\Gamma (\alpha +2)} \sum \limits _{n=2}^p\left[ \begin{array}{c} \tau _{n-1}^{\beta \left( \tau _{n-1}\right) }\times b_1\times g\left( \tau _{n-1}, x\left( \tau _{n-1}\right) \right) \\ -\tau _{n-2}^{\beta \left( \tau _{n-2}\right) }\times b_2 \times g\left( \tau _{n-2}, x\left( \tau _{n-2}\right) \right) \end{array}\right] \times A_5\nonumber \\&+\frac{\alpha }{A B(\alpha )} \frac{h^\alpha }{2 \Gamma (\alpha +3)} \sum \limits _{n=2}^p\left[ \begin{array}{c} b_3\times g\left( \tau _{n-1}, x\left( \tau _{n-1}\right) \right) \\ +b_4 \times g\left( \tau _{n-2}, x\left( \tau _{n-2}\right) \right) \end{array}\right] \times A_6. \end{aligned}$$

where $$A_5=(p-n+1)^{\alpha }(p-n+3+2\alpha )-(p-n)^{\alpha }(p-n+3+3 \alpha ),$$

$$A_6=\left[ \begin{array}{c} (p-n+1)^\alpha \left[ \begin{array}{c} 2(p-m)^2+(3 \alpha +10)(p-n) +2 \alpha ^2+9 \alpha +12 \end{array}\right] \\ -(p-n)^\alpha \left[ \begin{array}{c} 2(p-n)^2+(5 \alpha +10)(p-n)+6 \alpha ^2+18 \alpha +12 \end{array}\right] \end{array}\right]$$,

$$b_1=\left[ \frac{\beta \left( \tau _n\right) -\beta \left( \tau _{n-1}\right) }{h} \ln \tau _{n-1}+\frac{\beta \left( \tau _{n-1}\right) }{\tau _{n-1}}\right]$$, $$b_2=\left[ \frac{\beta \left( \tau _{n-1}\right) -\beta \left( \tau _{n-2}\right) }{h} \ln \tau _{n-2}+\frac{\beta \left( \tau _{n-2}\right) }{\tau _{n-2}}\right]$$,

$$b_3=\tau _n^{\beta \left( \tau _n\right) }\left[ \begin{array}{c} \frac{\beta \left( \tau _{n+1}\right) -\beta \left( \tau _n\right) }{h} \ln \tau _n +\frac{\beta \left( \tau _n\right) }{\tau _n} \end{array}\right] -2 \tau _{n-1}^{\beta \left( \tau _{n-1}\right) } \left[ \begin{array}{c} \frac{\beta \left( \tau _n\right) -\beta \left( \tau _{n-1}\right) }{h} \ln \tau _{n-1} +\frac{\beta \left( \tau _{n-1}\right) }{\tau _{n-1}} \end{array}\right]$$,

$$b_4=\tau _{n-2}^{\beta \left( \tau _{n-2}\right) }\left[ \begin{array}{c} \frac{\beta \left( \tau _{n-1}\right) -\beta \left( \tau _{n-2}\right) }{h} \ln \tau _{n-2} +\frac{\beta \left( \tau _{n-2}\right) }{\tau _{n-2}} \end{array}\right]$$.

## Variable order fractional derivative

In this section, the various type of variable order fractional derivatives for distinct chaotic attractor with new numerical schemes are presented.

In recent times, several novel chaotic systems have emerged, notably the Chen system, the generalized Lorenz system family, and the hyperbolic-type generalized Lorenz canonical form^46,47^. In reality, the Rossler system with a single wing exhibits topological and dynamic simplicity compared to the generalized Lorenz system, which features a double wing. It is evidently important to explore the creation of more intricate chaotic systems incorporating multi-scroll or multi-wing attractors, both in theoretical studies and practical experiments with engineering applications.

From both theoretical and practical perspectives, the preference is consistently for a lower-dimensional system characterized by a simpler algebraic structure yet one that exhibits more intricate topological features, including multi-wings, broader frequency bandwidths, and complex dynamics involving rich bifurcations. The question naturally arises as to whether a straightforward 3-D quadratic autonomous system can generate a genuine four-wing chaotic attractor with a wide frequency spectrum. This inquiry was addressed by Liu and Chen (2003), who initially suggested it was not feasible. However, in subsequent research, we demonstrate the existence of such a system, affirming that it can be implemented using physical circuits. Therefore, consider the 3-D four-wing smooth autonomous chaotic attractor of the Wang–Sun chaotic system:

5 $$\begin{aligned} {\mathbb {D}}^{\alpha (\tau )}u(\tau )=g_1=&x_1u(\tau )+x_2v(\tau )w(\tau )\nonumber \\ {\mathbb {D}}^{\alpha (\tau )}v(\tau )=g_2=&x_3u(\tau )+x_4v(\tau )-u(\tau )w(\tau )\nonumber \\ {\mathbb {D}}^{\alpha (\tau )}w(\tau )=g_3=&x_5u(\tau )v(\tau )+x_6w(\tau ). \end{aligned}$$

The employed method for obtaining (6) enables the reconstruction of asymptotically precise solutions for the partial differential equations (PDEs), derived from solutions of (6) with an error approaching zero. Specifically, the presence of chaotic trajectories in the Rucklidge system indicates the presence of chaotic trajectories in the PDEs. Therefore, our system serves as a legitimate representation of the PDEs within a parameter regime that has not undergone thorough examination. Nevertheless, this particular regime holds physical significance, as convection in a robust vertical magnetic field is known to occur in narrow rolls.

The Rucklidge chaotic system, distinct from the generalized Lorenz system, is a three-dimensional chaotic system resembling Lorenz. It serves as a representation of a dual-convection process. Therefore, the Rucklidge model characterizes the convection phenomenon in a horizontal layer of Boussinesq fluid, incorporating lateral constants to elucidate the convection dynamics, particularly at the juncture where chaotic solutions manifest. So consider the Rucklidge chaotic system:

6 $$\begin{aligned} {\mathbb {D}}^{\alpha (\tau )}u(\tau )=g_4=&-y_1u(\tau )+y_2v(\tau )-v(\tau )w(\tau )\nonumber \\ {\mathbb {D}}^{\alpha (\tau )}v(\tau )=g_5=&u(\tau )\nonumber \\ {\mathbb {D}}^{\alpha (\tau )}w(\tau )=g_6=&-w(\tau )+v(\tau )^2. \end{aligned}$$

The Rikitake system, derived through experimentation with a two-disk dynamo apparatus, represents a three-dimensional vector field. This system serves as a model for the geomagnetic field, providing insights into the observed irregular switch in its polarity. Characterized by a 3-dimensional Lorenz-type chaotic attractor centered around its two singular points, the Rikitake system lacks confinement within an ellipsoidal surface, distinguishing it from the Lorenz attractor. Geophysicists acknowledge that the Earth’s magnetic field has undergone numerous polarity reversals throughout its geological history. One common mechanical model employed to investigate these reversals is a two-disk dynamo system introduced by Rikitake^48^. In our Rikitake system (7), the notation $z_1$ denotes resistive dissipation, while the parameter $z_2$ signifies the variance in angular velocities between two dynamo discs.

The system comprises a pair of interconnected Faraday-disk dynamos, both identical and of the Bullard type. The dynamics of this system are governed by a set of three-dimensional nonlinear differential equations:

7 $$\begin{aligned} {\mathbb {D}}^{\alpha (\tau )}u(\tau )=g_7=&v(\tau )w(\tau )-z_1u(\tau )\nonumber \\ {\mathbb {D}}^{\alpha (\tau )}v(\tau )=g_8=&(w(\tau )-z_2)u(\tau )-z_1v(\tau )\nonumber \\ {\mathbb {D}}^{\alpha (\tau )}w(\tau )=g_9=&1-u(\tau )v(\tau ). \end{aligned}$$

**Table 1:** Parameter values and initial conditions for the chaotic systems.

Systems	Initial conditions	Parameter values
Wang–Sun	$u_0=-1,v_0=0,w_0=0.5$	$x_1=0.2,x_2=1,x_3=-0.01$, $x_4=-0.4,x_5=x_6=-1$
Rucklidge	$u_0=0.01,v_0=0.02,w_0=0.01$	$y_1=2,y_2=6.7$
Rikitake	$u_0=0.8,v_0=0.9,w_0=0.5$	$z_1=z_2=1$

The initial conditions and system parameter values for the Wang–Sun, Rucklidge, and Rikitake chaotic systems are shown in Table 1 for simple understanding.

### Remark 3.1

The integer-order dynamical system (7) is obviously unstable. However, the fractional order and variable order dynamical system is stable for any fractional and variable order $0<\alpha (\tau )<1$.

**Figure 1:**
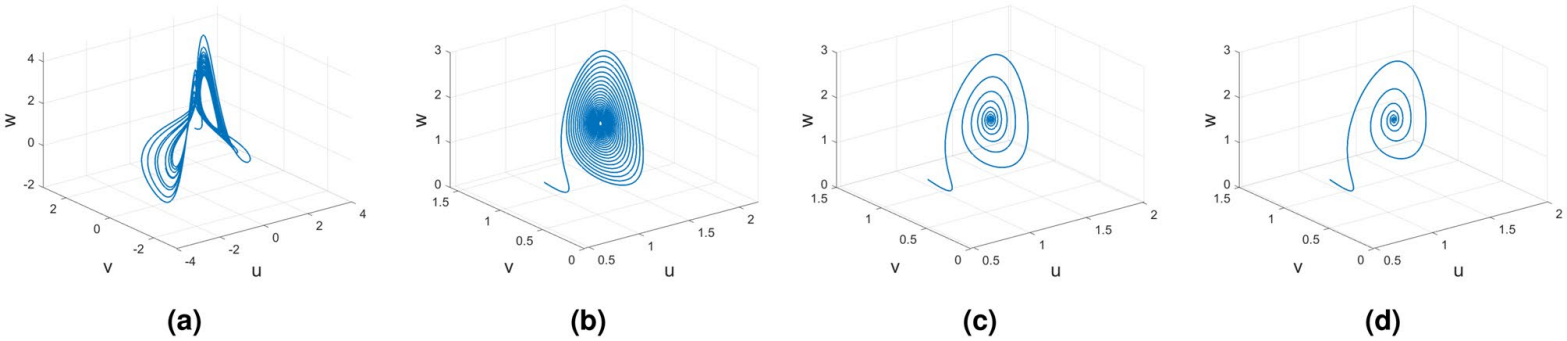
Integer and non-integer order Caputo derivative of Rikitake dynamical system.

Figure 1 presents the fractional order Caputo derivative. This figure represents the system’s periodic behavior for the fractional order $\alpha (tau)\in (0,1)$. Figure 1a is an integer-order unstable dynamical system, and Fig. 1b–d represents the Caputo derivative with various fractional orders.

### Numerical approach for Caputo fractional variable order derivative

In this subsection discuss the new type of numerical schemes for the variable order Caputo sense fractional derivative of Lorenz family attractions like Wang–Sun system, Rucklidge system, and Rikitake system.

The general solution can be applied in our chaotic system, as follows

$$\begin{aligned} u_{p+1}=&x_0+\frac{h^{\alpha (\tau )}}{\Gamma (\alpha (\tau )+1)} \sum \limits _{n=2}^p g_1\left( \tau _{n-2}, u_{n-2},v_{n-2},w_{n-2}\right) \left[ (p-n+1)^{\alpha (\tau )}-(p-n)^{\alpha (\tau )}\right] \\&+\frac{h^{\alpha (\tau )}}{\Gamma (\alpha (\tau )+2)} \sum \limits _{n=2}^p\left[ g_1\left( \tau _{n-1}, u_{n-1},v_{n-1},w_{n-1}\right) -g_1\left( \tau _{n-2}, u_{n-2},v_{n-2},w_{n-2}\right) \right] \times A_1\\&+\frac{h^{\alpha (\tau )}}{2 \Gamma (\alpha (\tau )+3)} \sum \limits _{n=2}^p\Big [g_1\left( \tau _n, u_n,v_n,w_n\right) -2g_1\left( \tau _{n-1}, u_{n-1},v_{n-1},w_{n-1}\right) \\&+g_1\left( \tau _{n-2}, u_{n-2},v_{n-2},w_{n-2}\right) \Big ] \times A_2,\\ v_{p+1}=&x_0+\frac{h^{\alpha (\tau )}}{\Gamma (\alpha (\tau )+1)} \sum \limits _{n=2}^p g_2\left( \tau _{n-2}, u_{n-2},v_{n-2},w_{n-2}\right) \left[ (p-n+1)^{\alpha (\tau )}-(p-n)^{\alpha (\tau )}\right] \\&+\frac{h^{\alpha (\tau )}}{\Gamma (\alpha (\tau )+2)} \sum \limits _{n=2}^p\left[ g_2\left( \tau _{n-1}, u_{n-1},v_{n-1},w_{n-1}\right) -g_2\left( \tau _{n-2}, u_{n-2},v_{n-2},w_{n-2}\right) \right] \times A_1\\&+\frac{h^{\alpha (\tau )}}{2 \Gamma (\alpha (\tau )+3)} \sum \limits _{n=2}^p\Big [g_2\left( \tau _n, u_n,v_n,w_n\right) -2g_2\left( \tau _{n-1}, u_{n-1},v_{n-1},w_{n-1}\right) \\&+g_2\left( \tau _{n-2}, u_{n-2},v_{n-2},w_{n-2}\right) \Big ] \times A_2,\\ w_{p+1}=&x_0+\frac{h^{\alpha (\tau )}}{\Gamma (\alpha (\tau )+1)} \sum \limits _{n=2}^p g_3\left( \tau _{n-2}, u_{n-2},v_{n-2},w_{n-2}\right) \left[ (p-n+1)^{\alpha (\tau )}-(p-n)^{\alpha (\tau )}\right] \\&+\frac{h^{\alpha (\tau )}}{\Gamma (\alpha (\tau )+2)} \sum \limits _{n=2}^p\left[ g_3\left( \tau _{n-1}, u_{n-1},v_{n-1},w_{n-1}\right) -g_3\left( \tau _{n-2}, u_{n-2},v_{n-2},w_{n-2}\right) \right] \times A_1\\&+\frac{h^{\alpha (\tau )}}{2 \Gamma (\alpha (\tau )+3)} \sum \limits _{n=2}^p\Big [g_3\left( \tau _n, u_n,v_n,w_n\right) -2g_3\left( \tau _{n-1}, u_{n-1},v_{n-1},w_{n-1}\right) \\&+g_3\left( \tau _{n-2}, u_{n-2},v_{n-2},w_{n-2}\right) \Big ] \times A_2. \end{aligned}$$

#### Variable order Wang–Sun chaotic system

Consider the Wang–Sun chaotic system of Caputo fractional variable order system

8 $$\begin{aligned} {}^C{\mathbb {D}}^{\alpha (\tau )}u(\tau )=&g_1(\tau ,u(\tau ),v(\tau ),w(\tau ))\nonumber \\ {}^C{\mathbb {D}}^{\alpha (\tau )}v(\tau )=&g_2(\tau ,u(\tau ),v(\tau ),w(\tau ))\nonumber \\ {}^C{\mathbb {D}}^{\alpha (\tau )}w(\tau )=&g_3(\tau ,u(\tau ),v(\tau ),w(\tau )). \end{aligned}$$

**Figure 2:**
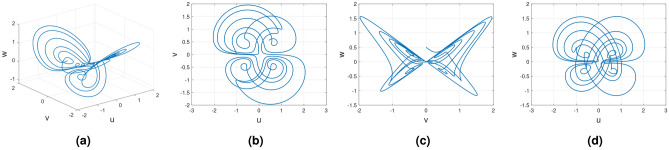
The Caputo fractional variable order derivative with chaotic behavior of Wang–Sun attractor.

In Fig. 2, represent the chaotic behavior of Wang–Sun attraction with variable order $\alpha (\tau )=\tanh (\tau +1)$. In Fig. 2a–d presents the chaotic nature of 3D state phase plane of *uvw*, *uv*- phase plane, *vw*- phase plane, *uw*- phase plane respectively.

#### Variable order Rucklidge chaotic system

Consider the Rucklidge chaotic system of Caputo fractional variable order system

9 $$\begin{aligned} {}^C{\mathbb {D}}^{\alpha (\tau )}u(\tau )=&g_4(\tau ,u(\tau ),v(\tau ),w(\tau ))\nonumber \\ {}^C{\mathbb {D}}^{\alpha (\tau )}v(\tau )=&g_5(\tau ,u(\tau ),v(\tau ),w(\tau))\nonumber \\ {}^C{\mathbb {D}}^{\alpha (\tau )}w(\tau )=&g_6(\tau ,u(\tau ),v(\tau ),w(\tau )). \end{aligned}$$

**Figure 3:**
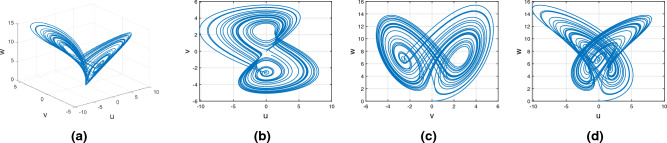
Rucklidge chaotic nature of the variable order Caputo fractional derivative of the state equation (*u*, *v*, *w*).

In Fig. 3, we represent the chaotic behavior of the variable-order Caputo fractional derivative of Rucklidge attraction with order $\alpha (\tau )=\tanh (\tau +1)$. Figure 3a–d present the chaotic nature of the 3D state phase planes of *uvw*, *uv*-phase plane, *vw*-phase plane, and *uw*-phase plane, respectively.

#### Variable order Rikitake chaotic system

Consider the Rikitake chaotic system of Caputo fractional variable order system

10 $$\begin{aligned} {}^C{\mathbb {D}}^{\alpha (\tau )}u(\tau )=&g_7(\tau ,u(\tau ),v(\tau ),w(\tau ))\nonumber \\ {}^C{\mathbb {D}}^{\alpha (\tau )}v(\tau )=&g_8(\tau ,u(\tau ),v(\tau ),w(\tau ))\nonumber \\ {}^C{\mathbb {D}}^{\alpha (\tau )}w(\tau )=&g_9(\tau ,u(\tau ),v(\tau ),w(\tau )). \end{aligned}$$

**Figure 4:**
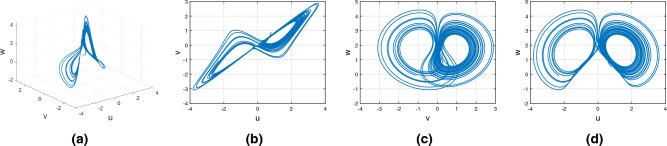
Chaotic behavior of the Rikitake attractor of variable order with Caputo fractional derivative.

In Fig. 4, study the chaotic behavior of the Rikitake attractor with order $\alpha (\tau )=\tanh (\tau +1)$ with variable order Caputo derivative sense. Figures 4a–d present the chaotic behavior of the 3D state phase planes of *uvw*, *uv*-phase plane, *vw*-phase plane, and *uw*-phase plane, respectively.

### Numerical approach for Caputo–Fabrizio fractional variable order derivative

The general solution can be applied in our chaotic system, as follows

$$\begin{aligned} u^{p+1}=&u^p+\frac{1-\alpha (\tau )}{M(\alpha (\tau ))}\left[ g_1\left( \tau _p, u\left( \tau _p\right) ,v\left( \tau _p\right) ,w\left( \tau _p\right) \right) -g_1\left( \tau _{p-1}, u\left( \tau _{p-1}\right) ,v\left( \tau _{p-1}\right) ,w\left( \tau _{p-1}\right) \right) \right] \\&+\frac{\alpha (\tau )}{M(\alpha (\tau ))}\left\{ \begin{array}{c} \frac{23}{12} g_1\left( \tau _p, u^p,v^p,w^p\right) h-\frac{4}{3} g_1\left( \tau _{p-1}, u^{p-1},v^{p-1},w^{p-1}\right) h\\ +\frac{5}{12} g_1\left( \tau _{p-2}, u^{p-2},v^{p-2},w^{p-2}\right) . \end{array}\right\} ,\\ v^{p+1}=&v^p+\frac{1-\alpha (\tau )}{M(\alpha (\tau ))}\left[ g_2\left( \tau _p, u\left( \tau _p\right) ,v\left( \tau _p\right) ,w\left( \tau _p\right) \right) -g_2\left( \tau _{p-1}, u\left( \tau _{p-1}\right) ,v\left( \tau _{p-1}\right) ,w\left( \tau _{p-1}\right) \right) \right] \\&+\frac{\alpha (\tau )}{M(\alpha (\tau ))}\left\{ \begin{array}{c} \frac{23}{12} g_2\left( \tau _p, u^p,v^p,w^p\right) h-\frac{4}{3} g_2\left( \tau _{p-1}, u^{p-1},v^{p-1},w^{p-1}\right) h\\ +\frac{5}{12} g_2\left( \tau _{p-2}, u^{p-2},v^{p-2},w^{p-2}\right) . \end{array}\right\} ,\\ w^{p+1}=&w^p+\frac{1-\alpha (\tau )}{M(\alpha (\tau ))}\left[ g_3\left( \tau _p, u\left( \tau _p\right) ,v\left( \tau _p\right) ,w\left( \tau _p\right) \right) -g_3\left( \tau _{p-1}, u\left( \tau _{p-1}\right) ,v\left( \tau _{p-1}\right) ,w\left( \tau _{p-1}\right) \right) \right] \\&+\frac{\alpha (\tau )}{M(\alpha (\tau ))}\left\{ \begin{array}{c} \frac{23}{12} g_3\left( \tau _p, u^p,v^p,w^p\right) h-\frac{4}{3} g_3\left( \tau _{p-1}, u^{p-1},v^{p-1},w^{p-1}\right) h\\ +\frac{5}{12} g_3\left( \tau _{p-2}, u^{p-2},v^{p-2},w^{p-2}\right) . \end{array}\right\} . \end{aligned}$$

#### Variable order Wang–Sun chaotic system

Consider the Wang–Sun chaotic system of Caputo–Fabrizio fractional variable order system

11 $$\begin{aligned} {}^{CF}{\mathbb {D}}^{\alpha (\tau )}u(\tau )=&g_1(\tau ,u(\tau ),v(\tau ),w(\tau ))\nonumber \\ {}^{CF}{\mathbb {D}}^{\alpha (\tau )}v(\tau )=&g_2(\tau ,u(\tau ),v(\tau ),w(\tau ))\nonumber \\ {}^{CF}{\mathbb {D}}^{\alpha (\tau )}w(\tau )=&g_3(\tau ,u(\tau ),v(\tau ),w(\tau )). \end{aligned}$$

**Figure 5:**
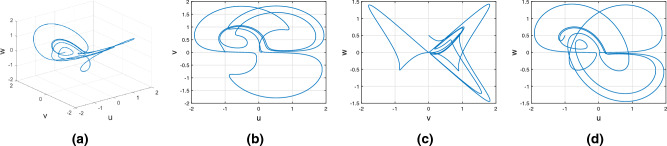
Numerical schemes for the variable order Caputo–Fabrizio fractional derivative of Wang–Sun system.

Figure 5 presents the chaotic behavior of Wang–Sun attraction with order $\alpha (\tau )=\tanh (\tau +1)$. Figures 5a–d present the variable-order Caputo–Fabrizio fractional derivative with the chaotic nature of the 3D state phase planes of *uvw*, *uv*-phase plane, *vw*-phase plane, and *uw*-phase plane, respectively. In this derivative, dynamical behavior is very slow when applied at $t = 200$. Moreover, to consider at time $t=400$, the dynamical nature is the same as the other fractional derivatives like Caputo, Caputo-Frabrizio, and Atangana–Baleanu–Caputo derivatives.

#### Variable order Rucklidge chaotic system

Consider the Rucklidge chaotic system of Caputo–Fabrizio fractional variable order system

12 $$\begin{aligned} {}^{CF}{\mathbb {D}}^{\alpha (\tau )}u(\tau )=&g_4(\tau ,u(\tau ),v(\tau ),w(\tau ))\nonumber \\ {}^{CF}{\mathbb {D}}^{\alpha (\tau )}v(\tau )=&g_5(\tau ,u(\tau ),v(\tau ),w(\tau ))\nonumber \\ {}^{CF}{\mathbb {D}}^{\alpha (\tau )}w(\tau )=&g_6(\tau ,u(\tau ),v(\tau ),w(\tau )). \end{aligned}$$

**Figure 6:**
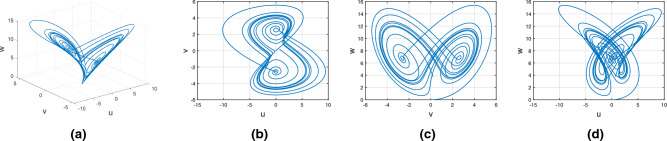
Caputo–Fabrizio variable order fractional derivative of Rucklidge system with chaotic attraction.

Figure 6 presents the chaotic behavior of Rucklidge attraction with order $\alpha (\tau )=\tanh (\tau +1)$. Figures 6a–d present the variable-order Caputo–Fabrizio fractional derivative with the chaotic nature of the 3D state phase planes of *uvw*, *uv*- phase plane, *vw*- phase plane, *uw*- phase plane respectively.

#### Variable order Rikitake chaotic system

Consider the Rikitake chaotic system of Caputo–Fabrizio fractional variable order system

13 $$\begin{aligned} {}^{CF}{\mathbb {D}}^{\alpha (\tau )}u(\tau )=&g_7(\tau ,u(\tau ),v(\tau ),w(\tau ))\nonumber \\ {}^{CF}{\mathbb {D}}^{\alpha (\tau )}v(\tau )=&g_8(\tau ,u(\tau ),v(\tau ),w(\tau))\nonumber \\ {}^{CF}{\mathbb {D}}^{\alpha (\tau )}w(\tau )=&g_9(\tau ,u(\tau ),v(\tau ),w(\tau )). \end{aligned}$$

**Figure 7:**
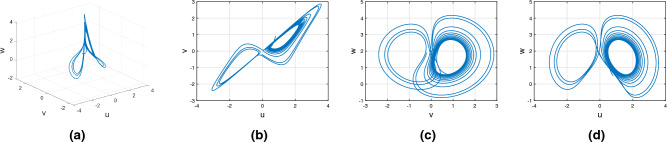
Chatoic nature for Variable order Caputo–Fabrizio derivative of Rikitake system.

Figure 7 presents the chaotic behavior of Rikitake attraction with order $\alpha (\tau )=\tanh (\tau +1)$. Figures 7a–d present the variable-order Caputo–Fabrizio fractional derivative with the chaotic nature of the 3D state phase planes of *uvw*, *uv*-phase plane, *vw*-phase plane, and *uw*-phase plane, respectively.

### Numerical approach for Atangana–Baleanu–Caputo fractional variable order derivative

In this section, discuss the Atangana–Baleanu–Caputo fractional variable order derivative of some distinct dynamical systems.

The general solution can be applied in our chaotic system, as follows,

$$\begin{aligned} u^{p+1}=&u(0)+\frac{1-\alpha (\tau )}{A B(\alpha (\tau ))} g_1\left( \tau _p, u^p,v^p,w^p\right) \\&+\frac{\alpha (\tau )h^{\alpha (\tau )}}{A B(\alpha (\tau )) \Gamma (\alpha (\tau )+1)} \sum \limits _{n=2}^p g_1\left( \tau _{n-2}, u^{n-2},v^{n-2},w^{n-2}\right) \left[ (p-n+1)^{\alpha (\tau )}-(p-n)^{\alpha (\tau )}\right] \\&+\frac{\alpha (\tau )h^{\alpha (\tau )}}{A B(\alpha (\tau )) \Gamma (\alpha (\tau )+2)} \sum \limits _{n=2}^p\left[ g_1\left( \tau _{n-1}, u^{n-1},v^{n-1},w^{n-1}\right) -g_1\left( \tau _{n-2}, u^{n-2},v^{n-2},w^{n-2}\right) \right] \times A_3\\&+\frac{\alpha (\tau )h^{\alpha (\tau )}}{2 A B(\alpha (\tau )) \Gamma (\alpha (\tau )+3)} \sum \limits _{n=2}^p\left[ \begin{array}{c} g_1\left( \tau _n, u^n,v^n,w^n\right) -2 g_1\left( \tau _{n-1}, u^{n-1},v^{n-1},w^{n-1}\right) \\ +g_1\left( \tau _{n-2}, u^{n-2},v^{n-2},w^{n-2}\right) \end{array}\right] \times A_4,\\ v^{p+1}=&v(0)+\frac{1-\alpha (\tau )}{A B(\alpha (\tau ))} g_2\left( \tau _p, u^p,v^p,w^p\right) \\&+\frac{\alpha (\tau )h^{\alpha (\tau )}}{A B(\alpha (\tau )) \Gamma (\alpha (\tau )+1)} \sum \limits _{n=2}^p g_2\left( \tau _{n-2}, u^{n-2},v^{n-2},w^{n-2}\right) \left[ (p-n+1)^{\alpha (\tau )}-(p-n)^{\alpha (\tau )}\right] \\&+\frac{\alpha (\tau )h^{\alpha (\tau )}}{A B(\alpha (\tau )) \Gamma (\alpha (\tau )+2)} \sum \limits _{n=2}^p\left[ g_2\left( \tau _{n-1}, u^{n-1},v^{n-1},w^{n-1}\right) -g_2\left( \tau _{n-2}, u^{n-2},v^{n-2},w^{n-2}\right) \right] \times A_3\\&+\frac{\alpha (\tau )h^{\alpha (\tau )}}{2 A B(\alpha (\tau )) \Gamma (\alpha (\tau )+3)} \sum \limits _{n=2}^p\left[ \begin{array}{c} g_2\left( \tau _n, u^n,v^n,w^n\right) -2 g_2\left( \tau _{n-1}, u^{n-1},v^{n-1},w^{n-1}\right) \\ +g_2\left( \tau _{n-2}, u^{n-2},v^{n-2},w^{n-2}\right) \end{array}\right] \times A_4,\\ w^{p+1}=&w(0)+\frac{1-\alpha (\tau )}{A B(\alpha (\tau ))} g_3\left( \tau _p, u^p,v^p,w^p\right) \\&+\frac{\alpha (\tau )h^{\alpha (\tau )}}{A B(\alpha (\tau )) \Gamma (\alpha (\tau )+1)} \sum \limits _{n=2}^p g_3\left( \tau _{n-2}, u^{n-2},v^{n-2},w^{n-2}\right) \left[ (p-n+1)^{\alpha (\tau )}-(p-n)^{\alpha (\tau )}\right] \\&+\frac{\alpha (\tau )h^{\alpha (\tau )}}{A B(\alpha (\tau )) \Gamma (\alpha (\tau )+2)} \sum \limits _{n=2}^p\left[ g_3\left( \tau _{n-1}, u^{n-1},v^{n-1},w^{n-1}\right) -g_3\left( \tau _{n-2}, u^{n-2},v^{n-2},w^{n-2}\right) \right] \times A_3\\&+\frac{\alpha (\tau )h^{\alpha (\tau )}}{2 A B(\alpha (\tau )) \Gamma (\alpha (\tau )+3)} \sum \limits _{n=2}^p\left[ \begin{array}{c} g_3\left( \tau _n, u^n,v^n,w^n\right) -2 g_3\left( \tau _{n-1}, u^{n-1},v^{n-1},w^{n-1}\right) \\ +g_3\left( \tau _{n-2}, u^{n-2},v^{n-2},w^{n-2}\right) \end{array}\right] \times A_4. \end{aligned}$$

#### Variable order Wang–Sun chaotic system

Consider the Wang–Sun chaotic system of Atangana–Baleanu–Caputo fractional variable order system

14 $$\begin{aligned} {}^{ABC}{\mathbb {D}}^{\alpha (\tau )}u(\tau )=&g_1(\tau ,u(\tau ),v(\tau ),w(\tau ))\nonumber \\ {}^{ABC}{\mathbb {D}}^{\alpha (\tau )}v(\tau )=&g_2(\tau ,u(\tau ),v(\tau ),w(\tau ))\nonumber \\ {}^{ABC}{\mathbb {D}}^{\alpha (\tau )}w(\tau )=&g_3(\tau ,u(\tau ),v(\tau ),w(\tau )). \end{aligned}$$

**Figure 8:**
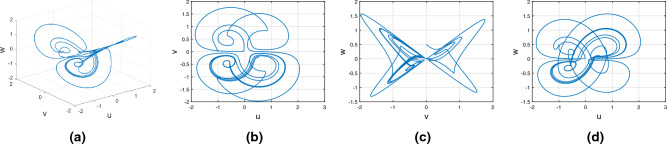
Dynamical behavior of variable order Atangana–Baleanu–Caputo derivative of Wang–Sun systems.

Figure 8 presents the chaotic behavior of Wang–Sun attraction with order $\alpha (\tau )=\tanh (\tau +1)$. Figures 8a–d present the variable-order Atangana–Baleanu–Caputo fractional derivative with the chaotic nature of the 3D state phase planes of *uvw*, *uv*-phase plane, *vw*-phase plane, and *uw*-phase plane, respectively.

#### Variable order Rucklidge chaotic system

Consider the Rucklidge chaotic system of Atangana–Baleanu–Caputo fractional variable order system

15 $$\begin{aligned} {}^{ABC}{\mathbb {D}}^{\alpha (\tau )}u(\tau )=&g_4(\tau ,u(\tau ),v(\tau ),w(\tau ))\nonumber \\ {}^{ABC}{\mathbb {D}}^{\alpha (\tau )}v(\tau )=&g_5(\tau ,u(\tau ),v(\tau ),w(\tau ))\nonumber \\ {}^{ABC}{\mathbb {D}}^{\alpha (\tau )}w(\tau )=&g_6(\tau ,u(\tau ),v(\tau ),w(\tau )). \end{aligned}$$

**Figure 9:**
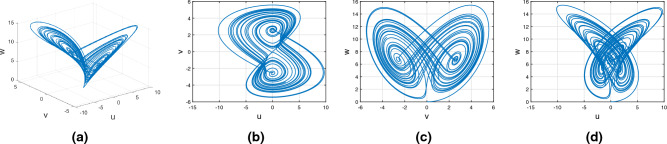
Dynamical behavior of the variable order Rucklidge system with Atangana–Baleanu– Caputo derivative sense.

Figure 9 presents the chaotic behavior of Rucklidge attraction with order $\alpha (\tau )=\tanh (\tau +1)$. Figures 9a–d present the variable-order Atangana–Baleanu–Caputo fractional derivative with the chaotic nature of the 3D state phase planes of *uvw*, *uv*-phase plane, *vw*-phase plane, and *uw*-phase plane, respectively.

#### Variable order Rikitake chaotic system

Consider the Rikitake chaotic system of Atangana–Baleanu–Caputo fractional variable order system

16 $$\begin{aligned} {}^{ABC}{\mathbb {D}}^{\alpha (\tau )}u(\tau )=&g_7(\tau ,u(\tau ),v(\tau ),w(\tau ))\nonumber \\ {}^{ABC}{\mathbb {D}}^{\alpha (\tau )}v(\tau )=&g_8(\tau ,u(\tau ),v(\tau ),w(\tau))\nonumber \\ {}^{ABC}{\mathbb {D}}^{\alpha (\tau )}w(\tau )=&g_9(\tau ,u(\tau ),v(\tau ),w(\tau )). \end{aligned}$$

**Figure 10:**
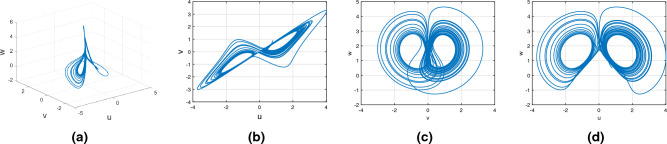
Dynamical nature of the Atangana–Baleanu–Caputo variable order derivative of Rikitake attractor.

Figure 10 presents the chaotic behavior of Rikitake attraction with order $\alpha (\tau )=\tanh (\tau +1)$. Figures 10a–d present the variable-order Atangana–Baleanu–Caputo fractional derivative with the chaotic nature of the 3D state phase planes of *uvw*, *uv*-phase plane, *vw*-phase plane, and *uw*-phase plane, respectively.

### Numerical approach for Atangana–Baleanu fractal fractional variable order derivative

In this subsection, the newly introduced integral and differential operator of various chaotic attractor are considered . That type of derivative is called the fractal and fractional derivative. Fractal fractional order derivatives offer a unique and powerful tool for modeling complex systems with non-local and memory-dependent behaviors, providing several advantages. Firstly, they offer a more nuanced representation of dynamical systems compared to traditional integer-order derivatives, capturing intricate features such as long-range correlations, self-similarity, and multi-fractal properties present in many natural phenomena. This enables more accurate and comprehensive modeling of complex systems across various fields, including physics, biology, finance, and engineering. Additionally, fractal fractional order derivatives exhibit superior adaptability to non-stationary and non-linear processes, making them particularly suitable for describing phenomena with evolving dynamics or irregular patterns. Moreover, their fractional nature facilitates the incorporation of memory effects, enabling the modeling of systems with memory-dependent behaviors, which are prevalent in many real-world applications.

The general solution can be applied in our chaotic system, as follows,

$$\begin{aligned} u^{p+1} =&u(0)+\frac{1-\alpha }{A B(\alpha )} \tau _p^{\beta \left( \tau _p\right) }\left[ \frac{\beta \left( \tau _{p+1}\right) -\beta \left( \tau _p\right) }{h} \ln \tau _p+\frac{\beta \left( \tau _p\right) }{\tau _p}\right] g_1\left( \tau _p, u\left( \tau _p\right) ,v\left( \tau _p\right) ,w\left( \tau _p\right) \right) \\&+\frac{\alpha }{A B(\alpha )} \frac{h^\alpha }{\Gamma (\alpha +1)} \sum \limits _{n=2}^p \tau _{p-2}^{\beta \left( \tau _{n-2}\right) }\left[ \frac{\beta \left( \tau _{n-1}\right) -\beta \left( \tau _{n-2}\right) }{h} \ln \tau _{n-2}+\frac{\beta \left( \tau _{n-2}\right) }{\tau _{n-2}}\right] \\&\times g_1\left( \tau _{n-2}, u\left( \tau _{n-2}\right) ,v\left( \tau _{n-2}\right) ,w\left( \tau _{n-2}\right) \right) \left[ (p-n+1)^\alpha -(p-n)^\alpha \right] \\&+\frac{\alpha }{A B(\alpha )} \frac{h^\alpha }{\Gamma (\alpha +2)} \sum \limits _{n=2}^p\left[ \begin{array}{c} \tau _{n-1}^{\beta \left( \tau _{n-1}\right) }\times b_1\times g_1\left( \tau _{n-1}, u\left( \tau _{n-1}\right) ,v\left( \tau _{n-1}\right) ,w\left( \tau _{n-1}\right) \right) \\ -\tau _{n-2}^{\beta \left( \tau _{n-2}\right) }\times b_2 \times g_1\left( \tau _{n-2}, u\left( \tau _{n-2}\right) ,v\left( \tau _{n-2}\right) ,w\left( \tau _{n-2}\right) \right) \end{array}\right] \times A_5\\&+\frac{\alpha }{A B(\alpha )} \frac{h^\alpha }{2 \Gamma (\alpha +3)} \sum \limits _{n=2}^p\left[ \begin{array}{c} b_3\times g_1\left( \tau _{n-1}, u\left( \tau _{n-1}\right) ,v\left( \tau _{n-1}\right) ,w\left( \tau _{n-1}\right) \right) \\ +b_4\times g_1\left( \tau _{n-2}, u\left( \tau _{n-2}\right) ,v\left( \tau _{n-2}\right) ,w\left( \tau _{n-2}\right) \right) \end{array}\right] \times A_6,\\ v^{p+1} =&v(0)+\frac{1-\alpha }{A B(\alpha )} \tau _p^{\beta \left( \tau _p\right) }\left[ \frac{\beta \left( \tau _{p+1}\right) -\beta \left( \tau _p\right) }{h} \ln \tau _p+\frac{\beta \left( \tau _p\right) }{\tau _p}\right] g_2\left( \tau _p, u\left( \tau _p\right) ,v\left( \tau _p\right) ,w\left( \tau _p\right) \right) \\&+\frac{\alpha }{A B(\alpha )} \frac{h^\alpha }{\Gamma (\alpha +1)} \sum \limits _{n=2}^p \tau _{p-2}^{\beta \left( \tau _{n-2}\right) }\left[ \frac{\beta \left( \tau _{n-1}\right) -\beta \left( \tau _{n-2}\right) }{h} \ln \tau _{n-2}+\frac{\beta \left( \tau _{n-2}\right) }{\tau _{n-2}}\right] \\&\times g_2\left( \tau _{n-2}, u\left( \tau _{n-2}\right) ,v\left( \tau _{n-2}\right) ,w\left( \tau _{n-2}\right) \right) \left[ (p-n+1)^\alpha -(p-n)^\alpha \right] \\&+\frac{\alpha }{A B(\alpha )} \frac{h^\alpha }{\Gamma (\alpha +2)} \sum \limits _{n=2}^p\left[ \begin{array}{c} \tau _{n-1}^{\beta \left( \tau _{n-1}\right) }\times b_1\times g_2\left( \tau _{n-1}, u\left( \tau _{n-1}\right) ,v\left( \tau _{n-1}\right) ,w\left( \tau _{n-1}\right) \right) \\ -\tau _{n-2}^{\beta \left( \tau _{n-2}\right) }\times b_2 \times g_2\left( \tau _{n-2}, u\left( \tau _{n-2}\right) ,v\left( \tau _{n-2}\right) ,w\left( \tau _{n-2}\right) \right) \end{array}\right] \times A_5\\&+\frac{\alpha }{A B(\alpha )} \frac{h^\alpha }{2 \Gamma (\alpha +3)} \sum \limits _{n=2}^p\left[ \begin{array}{c} b_3\times g_2\left( \tau _{n-1}, u\left( \tau _{n-1}\right) ,v\left( \tau _{n-1}\right) ,w\left( \tau _{n-1}\right) \right) \\ +b_4\times g_2\left( \tau _{n-2}, u\left( \tau _{n-2}\right) ,v\left( \tau _{n-2}\right) ,w\left( \tau _{n-2}\right) \right) \end{array}\right] \times A_6,\\ w^{p+1} =&w(0)+\frac{1-\alpha }{A B(\alpha )} \tau _p^{\beta \left( \tau _p\right) }\left[ \frac{\beta \left( \tau _{p+1}\right) -\beta \left( \tau _p\right) }{h} \ln \tau _p+\frac{\beta \left( \tau _p\right) }{\tau _p}\right] g_3\left( \tau _p, u\left( \tau _p\right) ,v\left( \tau _p\right) ,w\left( \tau _p\right) \right) \\&+\frac{\alpha }{A B(\alpha )} \frac{h^\alpha }{\Gamma (\alpha +1)} \sum \limits _{n=2}^p \tau _{p-2}^{\beta \left( \tau _{n-2}\right) }\left[ \frac{\beta \left( \tau _{n-1}\right) -\beta \left( \tau _{n-2}\right) }{h} \ln \tau _{n-2}+\frac{\beta \left( \tau _{n-2}\right) }{\tau _{n-2}}\right] \\&\times g_3\left( \tau _{n-2}, u\left( \tau _{n-2}\right) ,v\left( \tau _{n-2}\right) ,w\left( \tau _{n-2}\right) \right) \left[ (p-n+1)^\alpha -(p-n)^\alpha \right] \\&+\frac{\alpha }{A B(\alpha )} \frac{h^\alpha }{\Gamma (\alpha +2)} \sum \limits _{n=2}^p\left[ \begin{array}{c} \tau _{n-1}^{\beta \left( \tau _{n-1}\right) }\times b_1\times g_3\left( \tau _{n-1}, u\left( \tau _{n-1}\right) ,v\left( \tau _{n-1}\right) ,w\left( \tau _{n-1}\right) \right) \\ -\tau _{n-2}^{\beta \left( \tau _{n-2}\right) }\times b_2 \times g_3\left( \tau _{n-2}, u\left( \tau _{n-2}\right) ,v\left( \tau _{n-2}\right) ,w\left( \tau _{n-2}\right) \right) \end{array}\right] \times A_5,\\&+\frac{\alpha }{A B(\alpha )} \frac{h^\alpha }{2 \Gamma (\alpha +3)} \sum \limits _{n=2}^p\left[ \begin{array}{c} b_3\times g_3\left( \tau _{n-1}, u\left( \tau _{n-1}\right) ,v\left( \tau _{n-1}\right) ,w\left( \tau _{n-1}\right) \right) \\ +b_4\times g_3\left( \tau _{n-2}, u\left( \tau _{n-2}\right) ,v\left( \tau _{n-2}\right) ,w\left( \tau _{n-2}\right) \right) \end{array}\right] \times A_6. \end{aligned}$$

#### Variable order Wang–Sun chaotic system

Consider the Wang–Sun chaotic system of Atangana–Baleanu fractal fractional variable order system

17 $$\begin{aligned} {}^{ABFF}{\mathbb {D}}^{\alpha ,\beta (\tau )}u(\tau )=&g_1(\tau ,u(\tau ),v(\tau ),w(\tau ))\nonumber \\ {}^{ABFF}{\mathbb {D}}^{\alpha ,\beta (\tau )}v(\tau )=&g_2(\tau ,u(\tau ),v(\tau ),w(\tau))\nonumber \\ {}^{ABFF}{\mathbb {D}}^{\alpha ,\beta (\tau )}w(\tau )=&g_3(\tau ,u(\tau ),v(\tau ),w(\tau )). \end{aligned}$$

**Figure 11:**
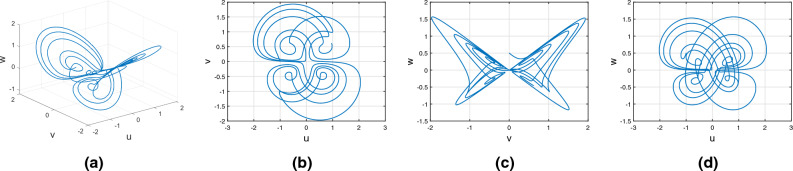
Atangana–Baleanu fractal and fractional derivative for chaotic nature of variable order Wang–Sun attraction.

Figure 11 represents the chaotic behavior of variable-order Wang–Sun attraction with order $\alpha =1$ and $\beta (\tau )=\tanh (\tau +1)$. Figures 11a–d present the variable-order Atangana–Baleanu fractal-fractional derivative with the chaotic nature of the 3D phase planes of *uvw*, *uv*-phase plane, *vw*-phase plane, and *uw*-phase plane, respectively.

#### Variable order Rucklidge chaotic system

Consider the Rucklidge chaotic system of Atangana–Baleanu Fractal fractional variable order system

18 $$\begin{aligned} {}^{ABFF}{\mathbb {D}}^{\alpha ,\beta (\tau )}u(\tau )=&g_4(\tau ,u(\tau ),v(\tau ),w(\tau ))\nonumber \\ {}^{ABFF}{\mathbb {D}}^{\alpha ,\beta (\tau )}v(\tau )=&g_5(\tau ,u(\tau ),v(\tau ),w(\tau ))\nonumber \\ {}^{ABFF}{\mathbb {D}}^{\alpha ,\beta (\tau )}w(\tau )=&g_6(\tau ,u(\tau ),v(\tau ),w(\tau )). \end{aligned}$$

**Figure 12:**
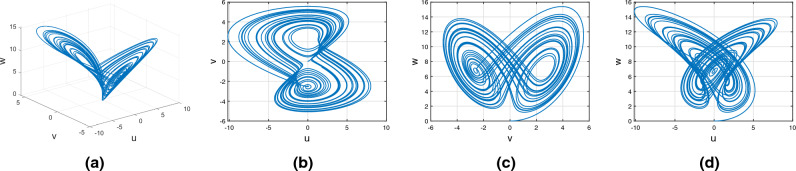
Variable order Atangana–Baleanu fractal and fractional derivative of Rucklidge chaotic behavior of (*u*, *v*, *w*) state equations.

Figure 12 represents the chaotic behavior of variable-order Rucklidge attraction with order $\alpha =1$ and $\beta (\tau )=\tanh (\tau +1)$. Figures 12a–d present the variable-order Atangana–Baleanu fractal-fractional derivative with the chaotic nature of the 3D phase planes of *uvw*, *uv*-phase plane, *vw*-phase plane, and *uw*-phase plane, respectively.

#### Variable order Rikitake chaotic system

Consider the Rikitake chaotic system of Atangana–Baleanu Fractal fractional variable order system

19 $$\begin{aligned} {}^{ABFF}{\mathbb {D}}^{\alpha ,\beta (\tau )}u(\tau )=&g_7(\tau ,u(\tau ),v(\tau ),w(\tau ))\nonumber \\ {}^{ABFF}{\mathbb {D}}^{\alpha ,\beta (\tau )}v(\tau )=&g_8(\tau ,u(\tau ),v(\tau ),w(\tau))\nonumber \\ {}^{ABFF}{\mathbb {D}}^{\alpha ,\beta (\tau )}w(\tau )=&g_9(\tau ,u(\tau ),v(\tau ),w(\tau )). \end{aligned}$$

**Figure 13:**
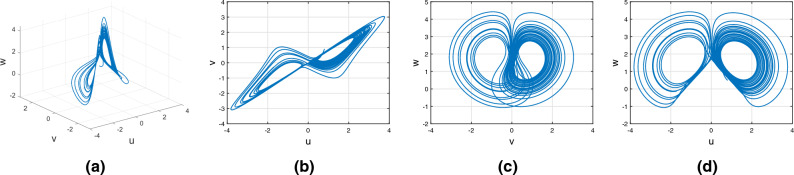
Numerical simulation of variable order Atangana–Baleanu fractal and fractional derivative of Rikitake equations.

Figure 13 represents the chaotic behavior of variable-order Rikitake attraction with order $\alpha =1$ and $\beta (\tau )=\tanh (\tau +1)$ and parameters $z_1=1,z_2=1$. Figures 13a–d present the variable-order Atangana–Baleanu fractal-fractional derivative with the chaotic nature of the 3D phase planes of *uvw*, *uv*-phase plane, *vw*-phase plane, and *uw*-phase plane, respectively.

## Stability analysis

In this section, we discussed the Hopf bifurcation and Lyapunov exponents of the fractional order Rikitake dynamical system. The corresponding linearized system is defined as follows with equilibrium points $(u^*,v^*,w^*)$.

20 $$\begin{aligned} {\mathbb {D}}^\alpha u(\tau )=&-z_1u(\tau )+w^*v(\tau )+v^*w(\tau ),\nonumber \\ {\mathbb {D}}^\alpha v(\tau )=&(w^*-z_2)u(\tau )-z_1v(\tau )+u^*w(\tau )\nonumber \\ {\mathbb {D}}^\alpha w(\tau )=&-v^*u(\tau )-u^*v(\tau ) \end{aligned}$$

In order to discuss the local stability results of system (20), we take the Laplace transform on both sides of (20).

21 $$\begin{aligned} t^\alpha U(t)-t^{\alpha -1}u(0)=&-z_1U(t)+w^*V(t)+v^*W(t),\nonumber \\ t^\alpha V(t)-t^{\alpha -1}v(0)=&(w^*-z_2)U(t)-z_1V(t)+u^*W(t),\nonumber \\ t^\alpha W(t)-t^{\alpha -1}w(0)=&-v^*U(t)-u^*V(t). \end{aligned}$$

Here, $U(t),\ V(t),\ W(t)$ are Laplace transform of $u(\tau ),\ v(\tau ),\ w(\tau )$. The above system (21) can be written as

$$\begin{aligned} \Delta (t)\times \begin{bmatrix} U(t)\\ V(t)\\ W(t) \end{bmatrix}=\begin{bmatrix} a_1(t)\\ a_2(t)\\ a_3(t) \end{bmatrix}, \end{aligned}$$

where

$$\begin{aligned} a_1(t)=&t^{\alpha -1}u(0),\\ a_2(t)=&t^{\alpha -1}v(0),\\ a_3(t)=&t^{\alpha -1}w(0), \end{aligned}$$

and

$$\begin{aligned} \begin{bmatrix} -z_1&{}w^*&{}v^*\\ w^*-z_2&{}-z_1&{}u^*\\ -v^*&{}-u^*&{}0 \end{bmatrix}. \end{aligned}$$

Then the characteristic matrix of the equilibrium point $\epsilon _1(1,1,z_1)$ when $z_2=0$. Therefore,

$$\begin{aligned} \Delta (t)=\begin{bmatrix} -z_1&{}z_1&{}1\\ z_1-z_2&{}-z_1&{}1\\ -1&{}-1&{}0 \end{bmatrix}. \end{aligned}$$

The characteristic equation at $\epsilon _1$ is given by

22 $$\begin{aligned} g(\lambda )=\lambda ^3 +2\,z_1 \,\lambda ^2 +{\left( z_1 \,z_2 +2\right) }\,\lambda +4\,z_1 -z_2. \end{aligned}$$

The fractional Hopf bifurcation will occur when complex roots of $g(\lambda )$ will cross into the cone $|\arg (\alpha )|<\frac{\pi \alpha }{2}$. Set $z_1=Z_h$, the Hopf critical value in the case $\lambda =re^{i\theta }$, where $\theta =\pm \frac{\pi \alpha }{2}$ will satisfy $g(\lambda )=0.$

Thus we get,

23 $$\begin{aligned}&{\textrm{e}}^{3\,\theta \,\textrm{i}} \,r^3 +2\,z_1 \,{\textrm{e}}^{2\,\theta \,\textrm{i}} \,r^2 +{\textrm{e}}^{\theta \,\textrm{i}} \,{\left( z_1 \,z_2 +2\right) }\,r+4\,z_1 -z_2=0,\nonumber \\&{\left( \cos \left( 3\,\theta \right) +\sin \left( 3\,\theta \right) \,\textrm{i}\right) }\,r^3 +2\,z_1 \,{\left( \cos \left( 2\,\theta \right) +\sin \left( 2\,\theta \right) \,\textrm{i}\right) }\,r^2 +{\left( \cos \left( \theta \right) +\sin \left( \theta \right) \,\textrm{i}\right) }\,{\left( z_1 \,z_2 +2\right) }\,r+4\,z_1 -z_2=0. \end{aligned}$$

Equating the real and imaginary parts in Eq. (23) we get,

24 $$\cos (3\theta )r^{3} + 2z_{1} \cos (2\theta )r^{2} + (z_{1} z_{2} + 2)\cos (\theta )r + 4z_{1} - z_{2} = 0,$$

25 $$\sin (3\theta )r^{3} + 2z_{1} \sin (2\theta )r^{2} + (z_{1} z_{2} + 2)\sin (\theta )r = 0.$$

As we are interested in non-zero solutions divided by *r* in Eq. (25), we get

26 $$\begin{aligned} \sin \left( 3\,\theta \right) \,r^2 +2\,z_1 \,\sin \left( 2\,\theta \right) \,r+2\,\sin \left( \theta \right) +z_1 \,z_2 \,\sin \left( \theta \right) =0. \end{aligned}$$

Then solve the above equation for *r*, we get

27 $$\begin{aligned} r_{1,2}=\frac{-2z_1\sin (2\theta )\pm \sqrt{\Lambda }}{2\sin (3\theta )}. \end{aligned}$$

where $$\Lambda =4z_1^2\sin ^2(2\theta )-4\sin (3\theta )(2+z_1z_2)\sin (\theta )$$.

Suppose to consider $\alpha >1/2$ and $z_1z_2>0$ we get $\Lambda >0$. Moreover, we consider $\alpha <1/2$ and $z_1z_2<$ we get $\Lambda <0$. From this case and using Eq. (25) we get,

28 $$\begin{aligned} r_1.r_2=\frac{z_1z_2\sin (\theta )}{\sin (3\theta )}<0. \end{aligned}$$

From the above equation implies that one of $r_1,r_2$ must be positive. Thus under the condition we are assured of one real root for (25). The corresponding roots of (22) are given as $\lambda _1=r_{1,2}e^{i\frac{\pi \alpha }{2}}, \lambda _2=r_{1,2}e^{-i\frac{\pi \alpha }{2}}$.

**Figure 14:**
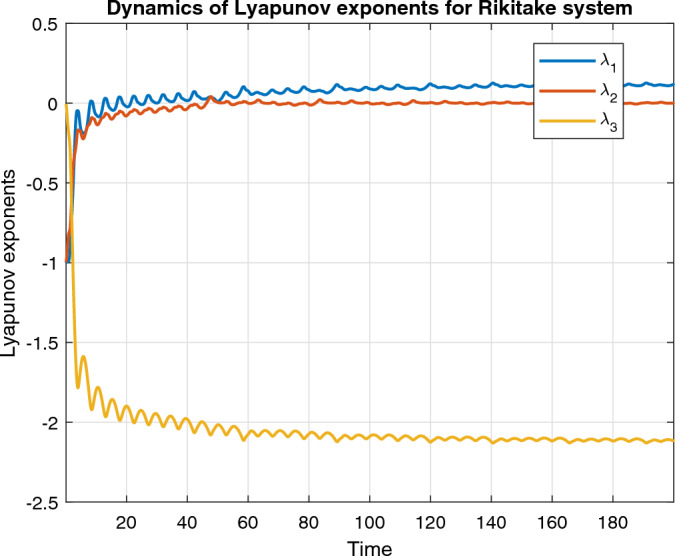
Lyapunov exponents of Rikitake system.

Figure 14, represent the Lyapunov exponents of the classical order Rikitake dynamical system.

## Comparative study

In this section, a comparative study about various aspects of the numerous derivatives, like the fractional variable order Caputo derivative, the Caputo Fabrizio derivative, the Atangana–Baleanu derivative, and finally the Atangana–Baleanu fractal and fractional derivative of distinct chaotic systems, are discussed.

### Wang–Sun dynamical system

In this subsection, consider the four useful derivatives in real-life scenarios for the Wang–Sun chaotic system.

The fractional derivatives are Caputo, Caputo–Fabrizio , Atangana–Baleanu–Caputo, and finally Atangana–Baleanu fractal and fractional derivatives applied in the Wang–Sun system.

**Figure 15:**
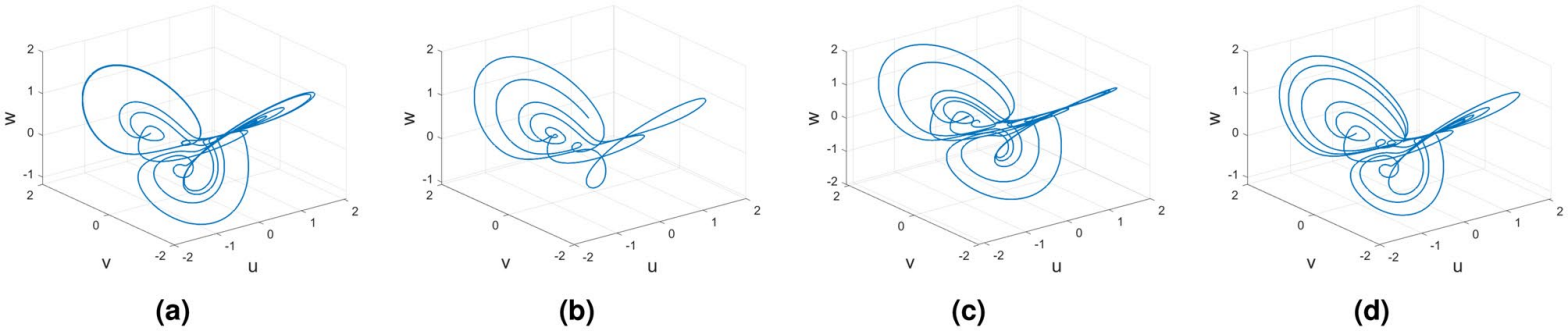
Wang–Sun dynamical system of variable order derivative with order $\alpha (\tau )=\frac{1}{(1+e^{-\tau })}$.

**Figure 16:**
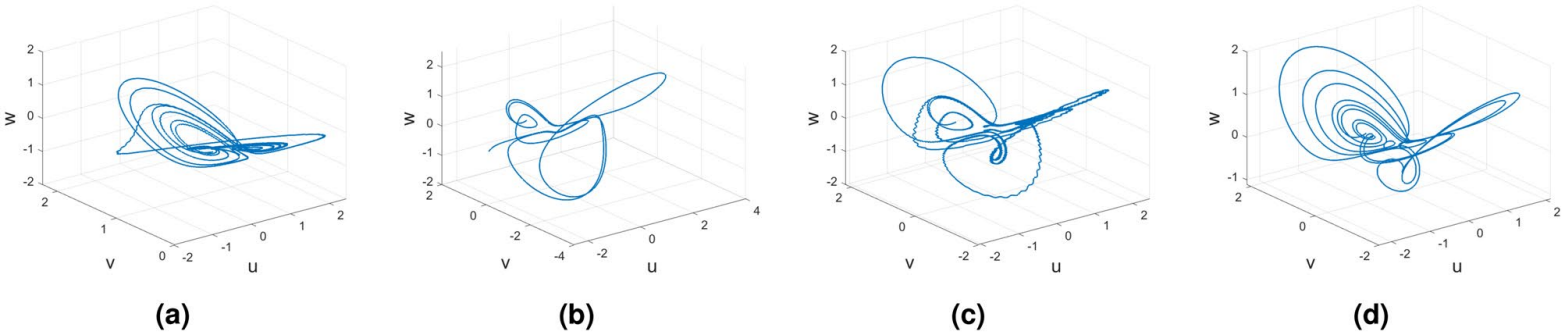
Wang–Sun dynamical system of variable order derivative with order $\alpha (\tau )=0.98+0.005\sin (\tau \pi /8)$.

**Figure 17:**
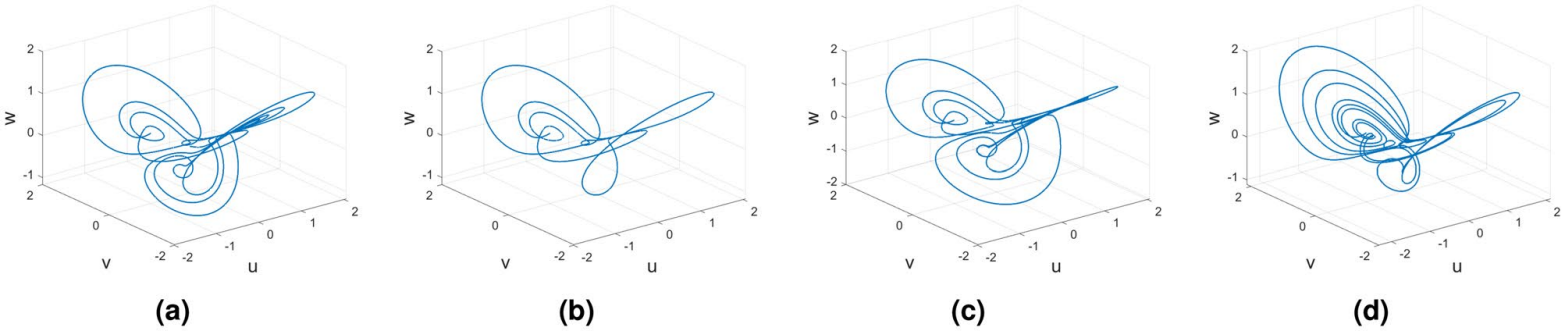
Wang–Sun dynamical system of integer order derivative with order $\alpha (\tau )=1$.

Figures 15, 16 and 17 show the various distinct variable-order derivatives that are applied and validated for the Wang–Sun dynamical systems. In Fig. 15, the variable-order fractional derivative is presented. In Fig. 15a, the Caputo fractional variable order derivative, and Fig. 15b, the Caputo–Fabrizio fractional variable order derivative, Fig. 15c shows the Atangana–Baleanu–Caputo derivative, and Fig. 15d displays the Atangana–Baleanu fractal-fractional variable order derivatives with order $\alpha =1$, $\beta (\tau )=\frac{1}{(1+e^{-\tau })}$, and then Fig. 15a–d represent the 3D chaotic behavior of the state equation (*u*, *v*, *w*) with variable order $\alpha (\tau )=\frac{1}{(1+e^{-\tau })}$. In Fig. 16, the variable-order fractional derivative is presented. Figures 16a–d represent the 3D chaotic behavior of the state equation (*u*, *v*, *w*) with variable order $\alpha (\tau )=0.98+0.005\sin (\tau \pi /8)$. In Fig. 17, the variable-order fractional derivative is presented. Figure 17a–d represent the 3D chaotic behavior of the state equation (*u*, *v*, *w*) with variable order $\alpha (\tau )=1$.

### Rucklidge dynamical system

**Figure 18:**
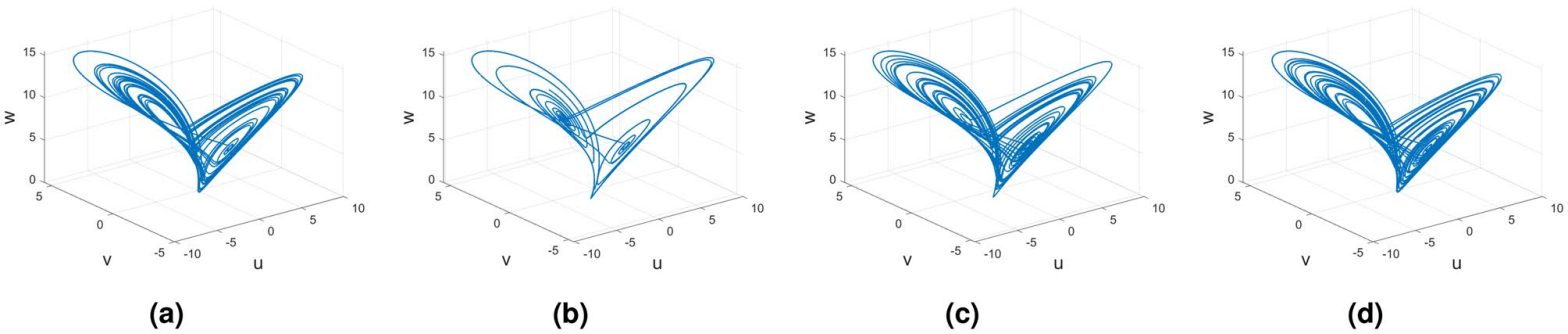
Rucklidge dynamical system of variable order derivative with order $\alpha (\tau )=\frac{1}{(1+e^{-\tau })}$.

**Figure 19:**
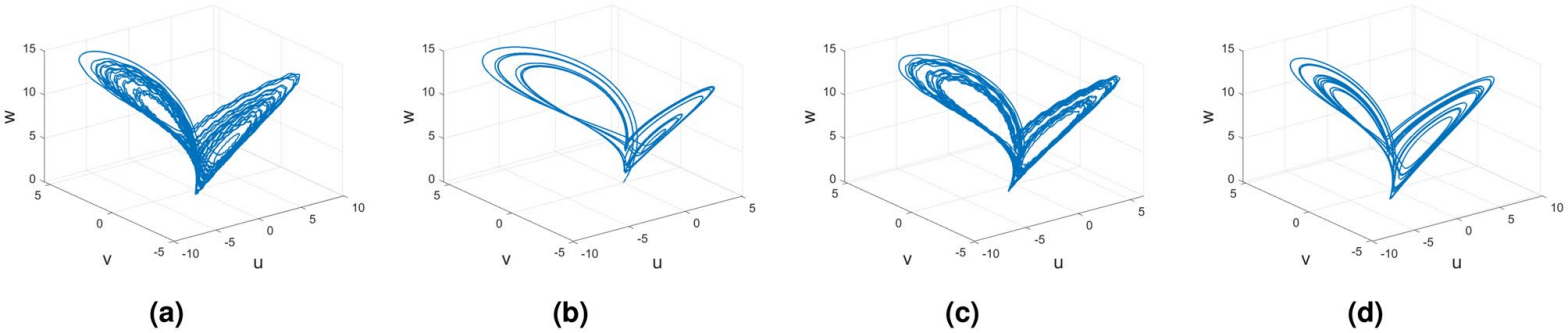
Rucklidge dynamical system of variable order derivative with order $\alpha (\tau )=0.98+0.005\sin (\tau \pi /8)$.

**Figure 20:**
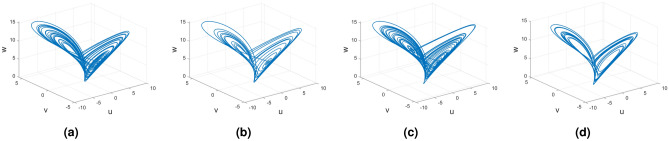
Rucklidge dynamical system of integer order derivative with order $\alpha (\tau )=1$.

In this subsection, consider the Rucklidge chaotic system to apply to four useful derivatives in real-life scenarios . Figures 18, 19 and 20 show the various distinct variable order derivatives that are applied and validated for the Rucklidge dynamical systems. In Fig. 18, the variable-order fractional derivative is presented. In Fig. 18a, the Caputo fractional variable order derivative, and Fig. 18b, the Caputo–Fabrizio fractional variable order derivative, Fig. 18c shows the Atangana–Baleanu–Caputo derivative, and Fig. 18d displays the Atangana–Baleanu fractal-fractional variable order derivatives with order $\alpha =1$, $\beta (\tau )=\frac{1}{(1+e^{-\tau })}$, and then Fig. 18a–d represent the 3D chaotic behavior of the state equation (*u*, *v*, *w*) with variable order $\alpha (\tau )=\frac{1}{(1+e^{-\tau })}$. In Fig. 19, the variable-order fractional derivative is presented. Figures 19a–d represent the 3D chaotic behavior of the state equation (*u*, *v*, *w*) with variable order $\alpha (\tau )=0.98+0.005\sin (\tau \pi /8)$. In Fig. 20, the variable-order fractional derivative is presented. Figures 20a–d represent the 3D chaotic behavior of the state equation (*u*, *v*, *w*) with variable order $\alpha (\tau )=1$.

### Rikitake dynamical system

In this subsection, consider the four useful derivatives in real-life scenarios for the Rikitake chaotic system.

**Figure 21:**
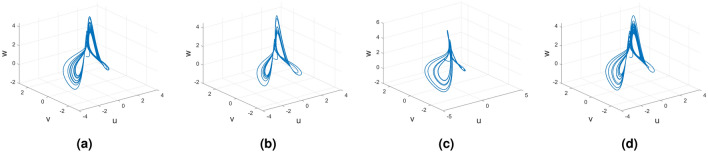
Rikitake dynamical system of variable order derivative with order $\alpha (\tau )=\frac{1}{(1+e^{-\tau })}$ and parameters $z_1=1,z_2=1$.

**Figure 22:**
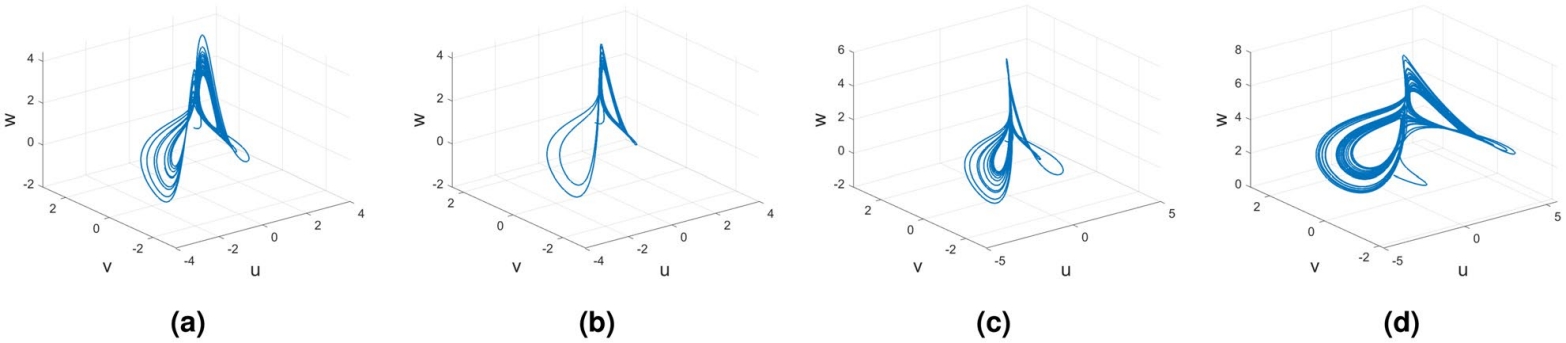
Rikitake dynamical system of variable order derivative with order $\alpha (\tau )=\tanh (\tau +1)$ and parameters $z_1=0.8,z_2=4$.

**Figure 23:**
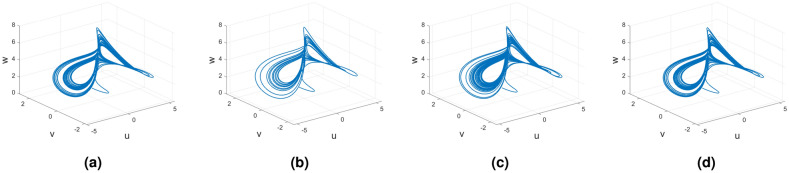
Rikitake dynamical system of integer order derivative with order $\alpha (\tau )=\frac{1}{(1+e^{-\tau })}$ and parameters $z_1=0.8,z_2=4$.

Figures 21, 22 and 23 show the various distinct variable-order derivatives that are applied and validated for the Rucklidge dynamical systems. In Fig. 21, the variable-order fractional derivative is presented. In Fig. 21a, the Caputo fractional variable order derivative, and Fig. 21b, the Caputo–Fabrizio fractional variable order derivative, Fig. 21c shows the Atangana–Baleanu–Caputo derivative, and Fig. 21d displays the Atangana–Baleanu fractal-fractional variable order derivatives with order $\alpha =1$, $\beta (\tau )=\frac{1}{(1+e^{-\tau })}$, and then Fig. 21a–d represent the 3D chaotic behavior of the state equation (*u*, *v*, *w*) with variable order $\alpha (\tau )=\frac{1}{(1+e^{-\tau })}$. In Fig. 22, the variable-order fractional derivative is presented. Figures 22a–d represent the 3D chaotic behavior of the state equation (*u*, *v*, *w*) with variable order $\alpha (\tau )=0.98+0.005\sin (\tau \pi /8)$. In Fig. 23, the variable-order fractional derivative is presented. Figure 23a–d represent the 3D chaotic behavior of the state equation (*u*, *v*, *w*) with variable order $\alpha (\tau )=1$.

## Conclusion

This study analyzed numerous variable orders for unique chaotic systems utilizing Newton’s polynomial interpolation, which was proposed for presenting computational solutions for fractional chaotic systems with power kernels with each variable order. In this work, the researchers established a numerical approach to deal with several chaotic challenges. We are certain of the method’s significant worth and the existence of several popular numerical schemes. The variable-order Adams-Bashforth approach has restrictions that are strengthened by the introduction of the alternative numerical method. The process is straightforward, effective, and precise. Moreover, a tiny discretization step is not required, compared to the Adams-Bashforth approach, which lowers computational effort. The new type of numerical approximation is applied and validated for the different chaotic systems with various variable-order fractional derivatives. In the future, this approximation will be applied to time-varying and time-independent delay systems in various real-life scenarios. Also, the chaotic behavior of the fractional dynamical systems using the Poincare map will be considered.

## Data Availability

The datasets used and/or analysed during the current study available from the corresponding author on reasonable request.
